# Virtual Trauma Interventions for the Treatment of Post-traumatic Stress Disorders: A Scoping Review

**DOI:** 10.3389/fpsyg.2020.562506

**Published:** 2020-11-13

**Authors:** Thiemo Knaust, Anna Felnhofer, Oswald D. Kothgassner, Helge Höllmer, Robert-Jacek Gorzka, Holger Schulz

**Affiliations:** ^1^Center for Mental Health, Bundeswehr Hospital Hamburg, Hamburg, Germany; ^2^Department of Pediatrics and Adolescent Medicine, Medical University of Vienna, Vienna, Austria; ^3^Department of Child and Adolescent Psychiatry, Medical University of Vienna, Vienna, Austria; ^4^Department of Applied Military and Operational Psychology, Military Police Command, Hanover, Germany; ^5^Department of Medical Psychology, University Hospital Hamburg-Eppendorf, Hamburg, Germany

**Keywords:** virtual reality exposure therapy, VRET, multi-modular motion-assisted memory desensitization and reconsolidation, 3MDR, action-centered exposure therapy, ACET, virtual trauma interventions, PTSD

## Abstract

Some post-traumatic stress disorder (PTSD) patients do not benefit from imaginal exposure therapy. One possible approach to reach such patients are virtual trauma interventions. Herein, a qualitative scoping review was conducted. Different types of virtual trauma exposure interventions were identified. For each type of virtual trauma exposure interventions it was examined in detail: (1) which *in sensu* trauma exposure approach serves as therapeutic framework, how it was transferred into virtual reality, and if it was manualized; (2) which hardware and software were used; (3) whether the influence of spatial and social presence on the efficacy of virtual trauma interventions have been measured, and (4) whether the efficacy of virtual trauma interventions for PTSD patients having imagination difficulties was evaluated. These research questions were analyzed qualitatively. Accordingly, an extensive literature search was conducted using the databases Web of Science, PsycINFO, LIVIVO, PTSDpubs, and PubMed for scientific articles published between January 2013 and July 2020. Only studies aimed to reduce PTSD symptoms using virtual trauma interventions were included. The literature search was not limited to a specific study design, treatment/intervention method, or a minimum sample size. Eighteen studies were identified, which reported three different virtual trauma intervention approaches, namely, virtual reality exposure therapy (VRET), multi-modular motion-assisted memory desensitization and reconsolidation (3MDR), and action-centered exposure therapy (ACET). Seven randomized controlled trials (RCTs), two pilot studies, and one case study were focused on VRET; while two RCTs, one pilot study, and three case studies focused on 3MDR, and two case studies on ACET. Regarding the first research question (1), the results show that VRET is based on prolonged exposure, aiming for a virtual re-creation of the patient's traumatic recounting. Several treatment protocols exist for VRET. 3MDR is based on eye movement desensitization and reprocessing, aiming to reduce the patient's avoidance behavior. In 3MDR patients walk toward individualized trauma-related symbolic images in a cave automatic virtual environment (CAVE). One treatment protocol exists for 3MDR. ACET is based on the inhibitory learning theory, aiming for active interactions with a virtual trauma-associated environment to alter the anxiety structure through new secondary inhibitory learning. One treatment protocol exists for ACET. For the second research question (2), the results indicate that all VRET studies used head-mounted displays (HMDs) with a virtual version of the Iraq/Afghanistan or the World Trade Center attacks, while 3MDR studies utilized two different versions of a CAVE with personalized trauma-related images, and the ACET studies used HMDs with virtual street scenarios. For the third research question (3), the results demonstrate that the influence of spatial or social presence on the efficacy of virtual trauma interventions was not examined in any of the included studies. Similarly, for the fourth research question (4), the results show that empirical evidence for the efficacy of virtual trauma interventions on PTSD patients having imagination difficulties was lacking. Therefore, such empirical studies are needed to fill these research gaps.

## Introduction

The main category of *in sensu* confrontation (or imaginal exposure) includes different interventions e.g., prolonged exposure (PE; Foa et al., 2007, 2019), eye movement desensitization and reprocessing (EMDR; Shapiro, 1989, 2018), or imagery rescripting and reprocessing therapy (IRRT, Schmucker and Köster, 2019). For the treatment of post-traumatic stress disorder (PTSD), PE and EMDR, are ranked as the oldest, best-examined, and most effective techniques (Foa et al., 2007; Powers et al., 2010; National Institute for Health Care Excellence, 2018; Schäfer et al., 2019). Despite the large empirical support for the efficacy of PE and EMDR not every patient benefits from these therapeutic approaches (examples for PE: Jaycox et al., 1998; Marks et al., 1998; Taylor et al., 2003; Foa et al., 2005, 2018; McDonagh et al., 2005; Schnurr et al., 2007; Pacella et al., 2011; Acierno et al., 2016; examples for EMDR: Marcus et al., 1997; Devilly et al., 1998; Power et al., 2002; Karatzias et al., 2011; Acarturk et al., 2016; Carletto et al., 2016). A recent meta-analysis showed a mean attrition rate of 22% for PE (included studies: *k* = 22) and 18% for EMDR (included studies: *k* = 21, Lewis et al., 2020). Furthermore, it remains unclear how many patients benefit from PE and EMDR in terms of remission and response rate. In some efficacy studies on PE, a remission rate of approximately 75% was reported (Marks et al., 1998; Taylor et al., 2003), whereas in other studies, it was approximately 40% (Foa et al., 1991; McDonagh et al., 2005; Schnurr et al., 2007; Fonzo et al., 2017). In a more recent study, the efficacy of mass (all sessions in 2 weeks, *N* = 110) was compared with spaced PE (all sessions in 8–15 weeks, *N* = 109), and remission rates of 54% and 51% were found (Foa et al., 2018). A similar pattern was observed for EMDR (Marcus et al., 1997; Carlson et al., 1998; Devilly and Spence, 1999; Högberg et al., 2007; Capezzani et al., 2013; Acarturk et al., 2016; Carletto et al., 2016). Here, efficacy studies revealed a remission rate of 36% (Devilly and Spence, 1999)−90% (Capezzani et al., 2013). This type of heterogeneity was also shown for the response rate of PE and EMDR (Foa et al., 1991; Devilly and Spence, 1999; Power et al., 2002; Schnurr et al., 2007; Asukai et al., 2010; Karatzias et al., 2011; Rauch et al., 2014). In efficacy studies on PE and EMDR, response rates of 40–90% (Foa et al., 1991; Rauch et al., 2014) and 27% (Devilly and Spence, 1999)−76% (Karatzias et al., 2011), respectively, were revealed (Supplementary Table 1 provides a more detailed overview of the cited studies and their operationalization of attrition, remission and response rate).

The reasons for the attrition, response and remission rates are multifactorial and not completely understood (Lewis et al., 2020). Some authors argue that for example, the trauma-type (Steenkamp et al., 2015; Wagenmans et al., 2018), psychiatric comorbidities (Van Minnen et al., 2012), high avoidance (Zoellner et al., 2011; Hundt et al., 2018; Van Gelderen et al., 2020) or insufficient imagination abilities (Jaycox et al., 1998) may explain whether patients can benefit from imaginal techniques. However, further work is required to verify these assumptions.

Nevertheless, this introductory overview provides evidence for the argument that not all patients benefit from PE and EMDR. To allow successful treatment for patients who are unable to benefit from classic trauma-focused guidelines, it is particularly important to use and examine the variety of treatment methods available in the context of psychotraumatology (Rizzo and Shilling, 2017; Carl et al., 2018; Kothgassner et al., 2019).

### Trauma Therapy and Virtual Reality

In general, trauma exposure can be explained by the emotional processing theory (Foa and Kozak, 1986), which presumes that the PTSD symptoms are based on pathological fear structures that are activated when patients are confronted with trauma-relevant information (Hembree et al., 2003). A reduction of symptoms requires a modification of the affective memory, enabling emotional processing such as the trauma-related information no longer evokes fear (Foa and Kozak, 1986). Therefore, imaginal exposure strives for mental engagement with the fear structure through confrontations (*in sensu* or *in vivo*) to achieve the habituation and extinction of an anxious reaction (Foa and Kozak, 1986; Foa et al., 2007). This can be practically achieved by the recounting of the patient's traumatic experience guided and encouraged by a therapist to imagine, narrate, and emotionally process the traumatic event (Foa et al., 2007; Leaman et al., 2013). However, this is particularly difficult for some patients, especially for those who cannot visualize the traumatic event or are not willing or prepared to do so because of memory gaps or severe avoidance behavior (e.g., an emotional defense mechanism) (Difede et al., 2007; Kehle-Forbes et al., 2016; Rizzo and Shilling, 2017; Loucks et al., 2018; Shulman et al., 2019). This can lead to a (premature) termination of treatment or to a less pronounced emotional reactivity when reporting the trauma, which negatively affects the treatment success (Jaycox et al., 1998; Difede et al., 2007; Foa et al., 2007; Cukor et al., 2015).

Therefore, studies have examined the possibility of incorporating virtual reality (VR) in trauma therapy to reach these patients (Rothbaum et al., 2001; Difede et al., 2007; Leaman et al., 2013; Vermetten et al., 2013; Cukor et al., 2015). There are various approaches to integrating VR in trauma therapy (Leaman et al., 2013; Vermetten et al., 2013). One kind of virtual trauma intervention is virtual reality exposure therapy (VRET) (Rothbaum et al., 1999, 2001). In VRET, head-mounted displays (HMDs) with preprogrammed virtual scenarios/environments are used to recreate the patients' traumatic experiences (Leaman et al., 2013; Rizzo and Shilling, 2017). Another way to use VR in trauma therapy is multi-modular motion-assisted memory desensitization and reconsolidation (3MDR) (Vermetten et al., 2013). 3MDR combines a treadmill with computer automatic virtual environments (CAVEs) to reduce PTSD patients' avoidance behavior (Vermetten et al., 2013; Van Gelderen et al., 2018). In 3MDR, it is assumed that walking toward a trauma-related symbolic representation of the patients' traumatic experience in a CAVE decreases avoidance behavior, increases therapy adherence, and reduces PTSD symptoms (Van Gelderen et al., 2018). The virtual environment can be customized through images selected by the patients (Van Gelderen et al., 2020).

From a cost-efficiency perspective, both approaches offer advantages and disadvantages. For example, an advantage of VRET is that the costs for HMDs are now less expensive and becoming more affordable, which makes VRET more obtainable in a clinical setting (Rizzo and Shilling, 2017; Kothgassner et al., 2019). However, a disadvantage of VRET is the high software cost (Rizzo and Shilling, 2017). To implement VRET in a clinical setting using IT, engineers program a virtual environment with preprogrammed and editable scenarios (Rizzo et al., 2005; Rizzo and Shilling, 2017). These scenarios are necessary tools that the therapist uses to customize the virtual environment based on the patient's recounting (Leaman et al., 2013). The generation of these scenarios is expensive and time-consuming. For example, Rizzo and Shilling (2017) mentioned a development time of 3 years. By contrast, for 3MDR, the hardware costs, for example, using CAVE, are still relatively high—ranging from $50,000–100,000 (Borrego et al., 2015; Coburn et al., 2017). Additionally, to conduct a 3MDR session, a technical operator and therapists are needed, which increases personnel costs (Vermetten et al., 2013; Van Gelderen et al., 2020). However, there is empirical evidence that 3MDR is a successful treatment for patients who cannot benefit from classic trauma-focused guidelines, which can reduce long-term therapy costs (Bisson et al., 2020; Van Gelderen et al., 2020). Nevertheless, the cost-efficiency aspect of virtual trauma intervention is not satisfactorily answered through research.

In addition to the general cost-efficiency debate, the most-examined virtual trauma intervention is VRET (Carl et al., 2018; Deng et al., 2019; Kothgassner et al., 2019). VRET uses HMD to offer a multi-sensory, anxiety-provoking, and trauma-specific virtual environment, which can be adjusted individually to the patients' own traumatic experience by adding or removing trauma-specific stimuli, leading to a controllable, repeatable, and emotionally engaging virtual trauma environment (Leaman et al., 2013; Ecrepont et al., 2016; Rizzo and Koenig, 2017; Rizzo and Shilling, 2017). This type of virtual re-creation is presumed to be helpful for patients with imagination difficulties to gain better access to their trauma-associated stimuli and memories (Jaycox et al., 1998; Difede et al., 2007; Leaman et al., 2013; Cukor et al., 2015; Rizzo and Shilling, 2017). In addition, VRET offers the advantage of gradually generating a standardized exposure and repeating it immediately *ad infinitum* (Leaman et al., 2013; Rizzo and Shilling, 2017; Kothgassner et al., 2019).

Several studies have been conducted to evaluate the efficacy of using VRET for PTSD (Difede et al., 2007; Ready et al., 2010; McLay et al., 2011; Miyahira et al., 2012). These early efficacy studies have revealed that VRET is superior to waitlist conditions with a medium to large effect size (Difede et al., 2007; Miyahira et al., 2012), although no significant differences in active controls have been shown e.g., present-centered therapy (PCT; Ready et al., 2010) or treatment as usual (TAU; McLay et al., 2011). Three recent meta-analyses have summarized the previous efficacy studies of VRET for PTSD (Carl et al., 2018; Deng et al., 2019; Kothgassner et al., 2019). Carl et al. (2018) identified five studies in which VRET was compared with the waitlist condition. The meta-analysis showed that VRET is superior to a waitlist with a medium effect size (hedge's *g* = 0.59, 95% CI [0.26, 0.92]). Similarly, Deng et al. (2019) found that VRET is superior to inactive controls (hedge's *g* = 0.56, 95% CI [0.27, 0.86], included studies: *k* = 5, *N* = 175), although there was no significant effect compared to the active controls (e.g., PE) (hedge's *g* = 0.01, 95% CI [-0.41, 0.44], included studies: *k* = 6, *N* = 239). Additionally, Kothgassner et al. (2019) revealed that VRET is superior to the waitlist control (hedge's *g* = 0.56, 95% CI [0.27, 0.86], included studies: *k* = 4, *N*_*VRET*_ = 54, N_Waitlist_ = 68), although there was no significant difference shown between VRET and the active controls (hedge's *g* = 0.25, 95% CI [−0.28, 0.79], included studies: *k* = 6, *N*_*VRET*_ = 100, N_activecontrols_ = 104).

These meta-analyses identified only two (Deng et al., 2019) and three studies (Kothgassner et al., 2019), which compared VRET to *in sensu* confrontation. Therefore, no meta-analyses so far have been conducted comparing VRET exclusively to *in sensu* confrontation (Carl et al., 2018; Deng et al., 2019; Kothgassner et al., 2019). Instead, *in sensu* confrontation was subsumed with different interventions (e.g., PCT) to active controls. Herein, the results show that VRET is superior to a waitlist, although no significant difference was shown between the active controls. However, recent reviews and meta-analyses have also indicated that the results have yet to be sufficiently confirmed statistically (Carl et al., 2018; Deng et al., 2019; Kothgassner et al., 2019). There have been very few randomized controlled studies (RCTs), and mostly small sample sizes have been used in the RCTs included in the meta-analyses (Carl et al., 2018; Deng et al., 2019; Kothgassner et al., 2019). Furthermore, these meta-analyses focused exclusively on VRET, which is one specific kind of virtual trauma exposure (Carl et al., 2018; Deng et al., 2019; Kothgassner et al., 2019). Therefore, less is known about other approaches that use VR in trauma therapy.

Except for the relatively well-established efficacy of VRET, the exact mechanism of virtual trauma exposure has yet to be fully clarified. It has generally been hypothesized that immersive technology enables the patient to experience a spatial presence (also known as the sense of being there in an artificial environment) (Wirth et al., 2007; Hartmann et al., 2016). Spatial presence, in turn, is presumed to be an essential precondition for activating a trauma-associated anxiety structure during VRET (Leaman et al., 2013; Vermetten et al., 2013; Cukor et al., 2015). In addition to spatial presence, it is also conceivable that social presence may influence the efficacy of VRET, particularly for interpersonal traumas such as sexual abuse or combat-related trauma (Ling et al., 2014; Oh et al., 2018). Social presence is described as “the sense of being with another” (Biocca et al., 2003, p. 456). It is essential for the user to experience a real social interaction with artificial intelligence-designed avatars (Lee et al., 2006; Oh et al., 2018). Without social presence, the sensation of being with another person in a virtual environment is “merely experienced as an artificial entity and not as a social being” (Oh et al., 2018, p. 2).

Moreover, in different studies in which VRET was used for other anxiety disorders, spatial presence was presumed to be an important factor influencing treatment success (Ling et al., 2014; Botella et al., 2017). For instance, Price and Anderson (2007) examined whether spatial presence during a virtual confrontation for patients with a fear of flying mediates treatment success. They showed that higher levels of spatial presence are related to higher in-session anxiety. However, they did not find that higher spatial presence is related to treatment success. Two more recent systematic reviews concluded that there is a significant correlation between spatial presence and anxiety during virtual confrontations (e.g., acrophobia: *r* = .35, *p* < 0.001, included correlations: *k* = 14; fear of animals: *r* = .50, *p* < 0.001, included correlations: *k* = 12 and fear of flying: *r* = .50, *p* < 0.001, included correlations: *k* = 12; Ling et al., 2014, p. 6) (Ling et al., 2014; Botella et al., 2017). However, it remains unclear whether the sense of presence is a precondition for treatment success, and the causal direction of the mean correlation between spatial presence and rated anxiety is still undetermined (Ling et al., 2014; Botella et al., 2017).

### Objectives of the Current Review

Prior reviews and meta-analyses have examined the efficacy of VRET in comparison to an active or inactive control group in anxiety disorders and PTSD (Botella et al., 2015; Carl et al., 2018; Deng et al., 2019; Kothgassner et al., 2019). It has yet to be summarized which other virtual trauma exposure intervention approaches exists, besides VRET. In more detail, it should be examined which *in sensu* trauma exposure approach serves as therapeutic framework for each identified type of virtual trauma exposure intervention, how it was transferred into VR, and if and how it is manualized. Furthermore, it is unclear which hardware and software are used for virtual trauma interventions and whether the experience of spatial or social presence is empirically assessed and influences the efficacy of a virtual confrontation. Finally, it is assumed that virtual trauma interventions are particularly effective for PTSD patients with imagination difficulties (Difede et al., 2007; Rizzo and Shilling, 2017; Loucks et al., 2018). However, it remains unclear whether previous studies have examined this assumption empirically. Therefore, it should be evaluated whether previous studies have empirically examined this assumption.

To identify as many virtual trauma interventions as possible, we conducted a qualitative scoping review. The literature search was not limited to a specific study design, treatment/intervention method or a minimal sample size. However, we only included studies with the goal of reducing PTSD symptoms with virtual trauma confrontations. More precisely, we defined the main characteristic of virtual trauma intervention to be an immersive technology. Technology is defined as immersive if it delivers “an inclusive, extensive, surrounding, and vivid illusion of reality” (Slater and Wilbur, 1997, p. 604). In general, HMDs and cave automatic virtual environments (CAVEs) meet these criteria and are considered immersive technologies (Cipresso et al., 2018, p. 4).

In conclusion, the overarching goal of the present review is to identify different types of virtual trauma exposure interventions and to examine for each identified type of virtual intervention (1) which *in sensu* trauma exposure approach serves as therapeutic framework for this type of virtual trauma exposure intervention, how it was transferred into VR (kind of virtual trauma exposure environment and procedure of virtual trauma exposure intervention), and if it is manualized; (2) assess which hardware and software are used; (3) examine whether the influence of spatial and social presence on the efficacy of virtual trauma interventions has been measured, and (4) evaluate whether these virtual trauma interventions are particularly effective for PTSD patients with imagination difficulties.

## Methods

### Scoping Review

We chose a scoping review with a qualitative synthesis as a suitable type of review particularly with regards to the accelerated development of immersive technology and the currently existing research gaps for virtual trauma interventions. The present scoping review followed current guidelines for scoping reviews (Pham et al., 2014; Peters et al., 2015; Tricco et al., 2018).

### Search Strategy

We performed a systematic literature search using the electronic databases Web of Science, PsycINFO, LIVIVO, PTSDpubs, and PubMed with the following search terms in German and English: (“VR” OR “Virtual Reality” OR “Virtuelle Realität”) AND (“PTBS” OR “PTSD” OR “Posttraumatische Belastungsstörung” OR “Posttraumatic Stress Disorder” OR “Post-traumatic Stress Disorder”). In 2013 the popularity of VR technology increased, based on the technological advances (the release of the Developer Kit of the Oculus Rift) (Luckerson, 2014; Cipresso et al., 2018). To increase the likelihood of identifying virtual trauma interventions, which should use modern VR technology, we conducted the literature search starting from January 2013 to August 2018 and performed an update of our search including the period from August 2018 to July 2020.

### Inclusion and Exclusion Criteria

Based on our research questions, we *a priori* defined the following inclusion and exclusion criteria: we included (1) studies which used any type of virtual trauma intervention with the goal to reduce PTSD symptoms; (2) studies which used immersive technology; (3) studies which used virtual exposure; (4) research in which PTSD was diagnosed according to ICD-10, DSM IV, DSM IV-R or DSM-5; (5) studies which examined patients between the ages of 18 and 65 years; (6) studies which were published from 2013 onwards, and (7) peer-reviewed articles.

We excluded (1) studies which used virtual trauma interventions for other purposes than for the treatment of PTSD^1^ e.g., basic research purpose and (2) studies which did not include original data (e.g., book chapters, reviews, study protocols, comments, etc.)^2^.

### Course of Evaluation of Suitability

**Figure 1:**
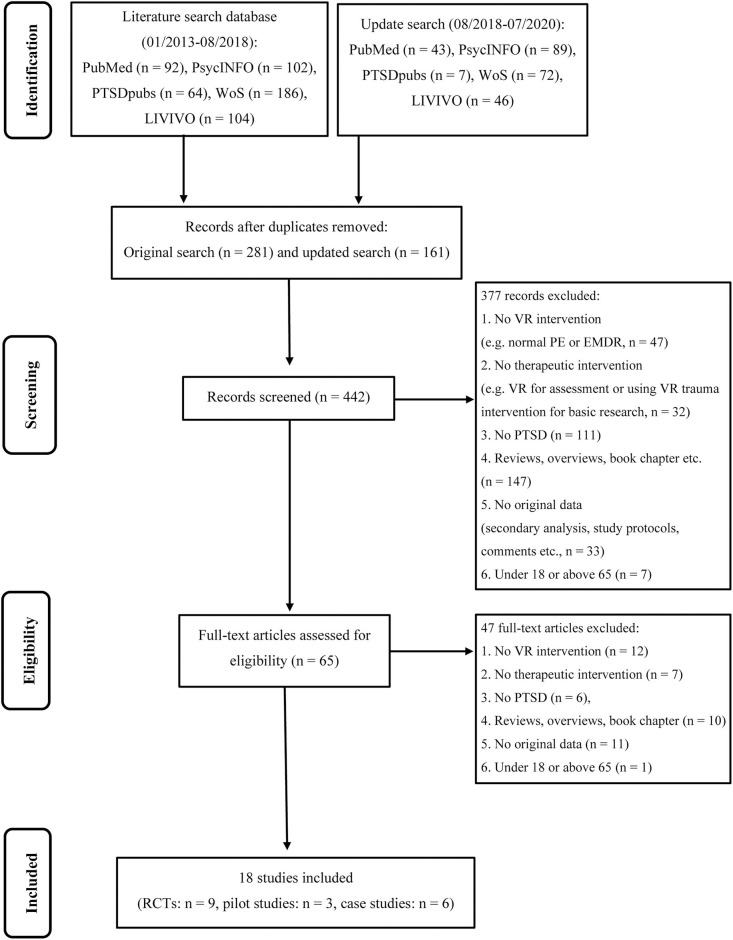
PRISMA flowchart of screening, exclusion, and inclusion criteria.

In the first step, we exported the title, abstract, year and authors of the identified literature into an Excel table and then removed duplicates. Two independent reviewers (VT, PS) read the titles and abstracts of each study. In case of disagreements, another reviewer (TK) was consulted, who decided whether the study met or did not meet the inclusion criteria. The PRISMA flowchart shows exclusions of studies at each stage of the process (see Figure 1).

### Data Extraction

A data extraction table was generated to answer the research questions. For the first research question, we extracted the type of virtual trauma exposure (e.g., VRET), the trauma-focused *in sensu* framework (e.g., PE or EMDR), how it was transferred into VR (e.g., virtual re-creation), and if it was manualized. If the studies used a manualized treatment protocol, we extracted in detail: the number of sessions and period of treatment time, the number and period of virtual exposure (in particular, it was examined whether there was a specific time limit of virtual exposure or did the studies conducted the virtual exposure until within-session habituation was attained), whether the treatment was supplemented with medication (if so, which medication was used), and whether at-home *in vivo* exposure exercises were included in the treatment protocol. For the second research question, the hardware and software used for the virtual trauma interventions were extracted. For the third research question, it was extracted whether and how the influence of spatial or social presence on the efficacy of virtual trauma interventions was measured. For the last one, we extracted whether and how the evaluation of efficacy for PTSD patients with imaginary problems were measured.

In addition, general descriptive variables were extracted. These included: authors, title, year, country of publication, the instrument for PTSD diagnosis (e.g., Diagnostic and Statistical Manual of Mental Disorders 5th Edition, DSM-5), the primary outcome variable, the time points of measurements (e.g., post-treatment, 3-month-follow-up, etc.), the effect sizes (if the authors did not reported the effect sizes, we extracted means, standard deviations and sample sizes for the respective studies. To recalculate the effect sizes we followed the procedure by Hedges and Olkin, 2014), the study design, the sample characteristic (military or civilian sample) and size, the drop-out rate, the average age of the sample, the gender distribution of the sample, and the trauma type. Furthermore, we extracted the objectives and main results of each included study.

### Data Synthesis

We provide a general overview of studies on virtual trauma intervention by calculating the overall descriptive statistics of the included studies (e.g., we calculated the sum of male patients over all the studies to obtain an overview of the gender distributions across all the studies). To analyze the research questions, we grouped the studies according to the identified types of virtual trauma interventions. For the first research question, we identified the *in sensu* trauma exposures that worked as therapeutic frameworks and qualitatively summarized how they were transferred to VR. To explore and compare the treatment protocols in further detail (the third part of the first research question), we tabulated and sorted the included studies according to the number of therapy sessions, time of each session, overall period of time, number of virtual exposures and duration of each. Thus, we examined whether the identified types of virtual trauma intervention followed the same procedure. After tabulating and sorting, we grouped the studies that were sufficiently similar and calculated descriptive statistics (percentage). Furthermore, we determined whether the researchers supplemented their treatment protocols with medication and at-home *in vivo* exercises. To summarize these results, we also calculated percentages. For the second research question, we calculated the percentage of the hardware and software used. The same procedure was followed for the third and fourth research questions.

## Results

### General Overview

We identified a total of 805 articles, of which 18 met the inclusion criteria. Of the 18 studies, nine (50%) (Difede et al., 2013; Rothbaum et al., 2014; Reger et al., 2016; Beidel et al., 2017a; McLay et al., 2017; Maples-Keller et al., 2018; Van‘t Wout et al., 2018; Bisson et al., 2020; Van Gelderen et al., 2020) were RCTs with a total of 483 patients (317 received VR treatment, 100 received an active treatment and 66 allocated to waitlist). Six (33.3%) (Vermetten et al., 2013; Arens, 2014; Kengne et al., 2018; Menelas et al., 2018; Nijdam and Vermetten, 2018; Van Gelderen et al., 2018) were case studies examined a total of 10 patients and three (16.6%) (Beidel et al., 2017b; Jetly et al., 2017; Loucks et al., 2018) were non-randomized single-arm trials subsumed to pilot studies with a total of 116 patients.

Ten studies (55.5%) (Difede et al., 2013; Arens, 2014; Rothbaum et al., 2014; Reger et al., 2016; Beidel et al., 2017a,b; McLay et al., 2017; Loucks et al., 2018; Maples-Keller et al., 2018; Van‘t Wout et al., 2018) were conducted in North America, three (16.6%) (Jetly et al., 2017; Kengne et al., 2018; Menelas et al., 2018) in Canada, four in the Netherlands (22.2%) (Vermetten et al., 2013; Nijdam and Vermetten, 2018; Van Gelderen et al., 2018, 2020) and one in Wales, United Kingdom (5.5%) (Bisson et al., 2020). Patients were predominantly male (92.7%), and the mean age ranged from 29 (Reger et al., 2016) – 51 (Nijdam and Vermetten, 2018). 15 (83.3%) studies included soldiers or veterans with combat-related PTSD, with one specializing on PTSD after a military sexual trauma (MST) (Loucks et al., 2018). Two studies (11.1%) reported results from civilian truck drivers after an accident (Kengne et al., 2018; Menelas et al., 2018) and one (5.5%) examined civilians with PTSD related to the attack on the World Trade Center (WTC) (Difede et al., 2013). Table 1 summarizes the descriptive analysis of the 18 articles.

**Table 1:** Descriptive characteristics of *k* = 18 included studies.

**References**	**Country**	**Instrument for PTSD diagnosis**	**Primary outcome variable**	**Study design**	**Sample and trauma type**	**Participants and drop-out**	**Intervention**	**Time points of measurements and main results**
Arens (2014)	USA	DSM-IV-TR	CAPS	Case study	War veteran with past Iraq and Afghanistandeployment. Combat-related PTSD	Participant: *N* = 1, drop-out: n.a., age: 45, gender: 1 (100%) male	VRET with TMT	Measurements: Pre, post, and 3 month-follow-up Effect size: n.a. Summary: Clinically significant decreases in overall PTSD symptoms. The symptom decrease was maintained at the 3 month-follow-up
Beidel et al. (2017a)	USA	DSM-IV	CAPS	RCT	War veterans and active duty personnel with past Iraq and Afghanistan deployment Combat-related PTSD symptoms	Total participants: *N* = 92, drop-out: 43 (41.3%), mean age: 35.4, gender: 86 (93.4%) males VR treatment group (VRET with TMT): *n* = 49, drop-out: 18 (36%), mean age: 37.6, gender: 45 (91%) males Active control group (VRET with psychoeducation): *n* = 43, drop-out: 25 (58%), mean age: 33.2, gender: 41 (95%) males	VRET with TMT vs. VRET with psychoeducation	Measurements: Pre, post, 3 and 6 month-follow-up Effect size (CAPS): Hedges' *g*_post_ = −0.36^#^ (favoring active control group) Hedges' *g*_3month_ = −0.47^#^ Hedges' *g*_6month_ = −0.62^#^ Summary: Significant decrease on the CAPS for both treatment groups. According to the authors there were no significant differences between the groups (p. 70). Treatment gains were maintained at 6 month follow up
Beidel et al. (2017b)	USA	DSM-IV-TR	CAPS	Pilot study	War veterans and active duty personnel with past Iraq and Afghanistan deployment Combat- related PTSD symptoms	Total participants: *N* = 112, drop-out: 10 (9.8%), mean age: 37.1, gender: 97 (95%) males	VRET with TMT	Measurements: Pre, post, 3 and 6 month-follow-up Effect size (CAPS): Cohen's *d*_pre−post_ = 2.06 Cohen's *d*_pre−3month_ = n.r. Cohen's *d*_pre−6month_ = n.r. Summary: Significant decrease on CAPS from pre to post-treatment. Treatment gains were maintained at 6 month follow up
Difede et al. (2013)	USA	DSM-IV	CAPS	RCT	Civilians, who had PTSD symptoms following exposure to the WTC attacks.	Total participants: *N* = 25, drop-out: 3 (12%), mean age: 45.7, gender: 19 (76%) males VR treatment group (VRET with DCS): *n* = 13, drop-out: 0 (0%), mean age: 47.7, gender: 8 (61.5%) males Active control group (VRET with placebo): *n* = 12, drop-out: 3 (25%), mean age: 43.7, gender: 11 (91.6%) males	VRET with DCS vs. VRET with placebo	Measurements: Pre, post, and 6 month-follow-up Effect size (CAPS): Hedges' *g*_post_ = 0.68 (favoring VRET with DCS) Hedges' *g*_6month_ = 1.13 Summary: Significant decrease on the CAPS for both treatment groups. At 6 month-follow-up VRET with DCS were superior to active control group
Loucks et al. (2018)	USA	DSM-5	CAPS	Pilot study	Military veterans with PTSD symptoms due military sexual trauma (MST)	Total participants: *N* = 15, drop-out: 6 (40%), mean age: 46, gender: 11 (73.4%) females	VRET	Measurements: Pre, post, and 3 month-follow-up Effect size (CAPS): Cohen's *d*_pre−post_ = 1.11 Cohen's *d*_pre−3month_ = n.r. Summary: This effect maintained at 3 month-follow-up. Results suggest that VRET is a potential treatment for MST related PTSD
Maples-Keller et al. (2018)	USA	DSM-5	CAPS	RCT	War veterans and active duty personnel with past Iraq and Afghanistan deployment Combat- related PTSD symptoms	Total participants: *N* = 27, drop-out: 3 (12%), mean age: 35.4, gender: 27 (100%) males VR treatment group (VRET with dexamethasone): *n* = 13, drop-out: 0 (0%), mean age: n.r., gender: 13 (100%) males Active control group (VRET with placebo): *n* = 14, drop-out: 3 (25%), mean age: n.r., gender: 14 (100%) males	VRET with dexamethasone vs. VRET with placebo	Measurements: Pre and post Effect size (CAPS): Combined sample Cohen's *d*_pre−post_ = n.r. Summary: Significant decrease on the CAPS for post-treatment but no significant differences between groups
McLay et al. (2017)	USA	DSM-IV	CAPS	RCT	Active duty military members with past Iraq and Afghanistan deployment Combat- related PTSD symptoms	Total participants: *N* = 81, drop-out: 7 (8%), mean age: 32.5, gender: 78 (96.3%) males VR treatment group (VRET with immersive technology): *n* = 43, drop-out: 7 (16%), mean age: 33, gender: 40 (93%) males Active control group (VRET with non-immersive technology): *n* = 38, drop-out: 0 (0%), mean age: 32, gender: 38 (100%) males	VRET with immersive technology vs. VRET with non-immersive technology	Measurements: Pre, post, and 3 month-follow-up Effect size (CAPS): Hedges' *g*_post_ = −0.33^#^ (favoring VRET with non-immersive technology) Hedges' *g*_3month_ = 0.15^#^ (favoring VRET with immersive technology) Summary: Significant decrease on the CAPS maintained over 3 month-follow-up. No significant differences between groups were found
Reger et al. (2016)	USA	DSM-IV-TR	CAPS	RCT	Active duty military members with past Iraq and Afghanistan deployment Combat- related PTSD symptoms	Total participants: *N* = 162, drop-out: 54 (33%), mean age: 30.2, gender: 156 (96.3%) males VR treatment group (VRET): *n* = 54, drop-out: 14 (25%), mean age: 29.5, gender: 52 (96%) males Active control group (prolonged exposure): *n* = 54, drop-out: 12 (22%), mean age: 30.8, gender: 51 (94%) males Control group (waitlist): *n* = 54, drop-out: 7 (12%), mean age: 30.3, gender: 53 (98%) males	VRET vs. PE vs. waiting list	Measurements: Pre, post, 3 and 6 month-follow-up Effect size (CAPS): VRET vs. PE: Hedges' *g*_post_ = −0.38^#^ (favoring PE) VRET vs. Control: Hedges' *g*_post_ = −0.39^#^ (favoring VRET) PE vs. Control: Hedges' *g*_post_ = 0.84^#^ (favoring PE) VRET vs. PE: Hedges' *g*_3month_ = −0.63^#^ (favoring PE) VRET vs. PE: Hedges' *g*_6month_ = −0.33^#^ (favoring PE) Summary: According to the authors VRET and PE were superior to wait-list at post-treatment. At 3- and 6 month-follow-up VRET were inferior to PE
Rothbaum et al. (2014)	USA	DSM-IV-TR	CAPS	RCT	War veterans with Iraq and Afghanistan deployment Combat- related PTSD symptoms	Total participants: *N* = 156, drop-out: 59 (37%), mean age: 35.1, gender: 148 (94%) males VR treatment group (VRET with DCS): *n* = 53, drop-out: 25 (47%), mean age: 34.9, gender: 49 (92%) males Active control group (VRET with Alprazolam): *n* = 50, drop-out: 15 (30%), mean age: 36.2, gender: 49 (98%) males Control group (VRET with placebo): *n* = 53, drop-out: 19 (35%), mean age: 34.3, gender: 50 (94%) males	VRET with DCS vs. VRET with Alprazolam vs. VRET with Placebo	Measurements: Pre, post, 3, 6, and 12 month-follow-up Effect size: n.r. and n.a.^#^ Summary: All groups decreased significantly on the CAPS. The effect maintained over 12 months-follow-up. At post-treatment there was no significant difference between D-cycloserin and placebo group on the CAPS. However, there was a significant difference favoring placebo over alprazolam regarding the CAPS at post-treatment
Van‘t Wout et al. (2018)	USA	DSM-5	PCL-5	RCT	War veterans with Iraq and Afghanistan deployment Combat-related PTSD symptoms	Total participants: *N* = 12, drop-out: n.r., mean age: 40.5, gender: 12 (100%) males VR treatment group (VRET with tDCS): *n* = n.r., drop-out: n.r., mean age: n.r., gender: n.r. Active control group (VRET with sham tDCS): *n* = n.r., drop-out: n.r., mean age: n.r., gender: n.r.	VRET with tDCS vs. VRET with sham tDCS	Measurements: Pre, post, and 1 month-follow-up Effect size (PCL-5): Hedges' *g*_post_ = 0.20^#^ (favoring VRET with tDCS) Cohen's *d*_1month_ = 0.37 Summary: Both groups demonstrated significant reductions in PCL scores. There were no significant differences between groups at post time measurement, but VRET with tDCS were superior to VRET sham tDCS at 1 month-follow-up
Bisson et al. (2020)	United Kingdom, Wales	DSM-5	CAPS-5	RCT	Military veterans with treatment- resistant and combat-related PTSD Treatment- resistance were defined as persisting PTSD diagnosis following a trauma-focused therapy	Total participants: *N* = 42, drop-out: 6 (14.2%), mean age: 42.1, gender: 42 (100%) males VR treatment group (3MDR): *n* = 21, drop-out: 4 (19%), mean age: 40.2, gender: 21 (100%) males Control group (waitlist): *n* = 21, drop-out: 2 (4.7%), mean age: 44.0, gender: 21 (100%) males	3MDR vs. waiting list (participants allocated to waiting list received 3MDR after a delay of 12 weeks)	Measurements: Pre, 12, and 26 weeks after randomization Effect size (CAPS-5): Cohen's *d*_12weeks_ = 0.65 (favoring 3MDR) Summary: Results indicated that 3MDR reduces PTSD symptoms in Veterans with treatment-resistant and combat-related PTSD. The authors conclude that phase III trials with larger sample sizes are warranted
Jetly et al. (2017)	Canada	n.r.	PCL-5	Pilot study	Soldiers with treatment-resistant and combat- related PTSD Treatment-resistant were defined as patients “who were non responders to at least one type of trauma-focused. psychotherapy” (p. 1)	Total participants: *N* = 8, drop-out: 3 (37.5%), mean age: n.r., gender: n.r.	3MDR	Measurements: Pre and post PCL-5 effect size (PCL-5): Cohen's *d*_pre−post_ = n.r. Summary: Modest therapy gains for patients with treatment-resistant and combat-related PTSD. The authors use these preliminary results to conduct larger randomized controlled trials (p. 2)
Nijdam and Vermetten (2018)	Netherlands	n.r.	n.r.	Case study	War veteran with Afghanistan deployment Treatment- resistant and combat-related PTSD	Participant: *N* = 1, drop-out: n.a., age: 51, gender: 1 (100%) male	3MDR	Measurements: n.r. Effect size: n.a. Summary: Positive treatment results for a single patient with treatment-resistant and combat-related PTSD
Van Gelderen et al. (2018)	Netherlands	n.r.	PCL-5	Case study	War veterans with French foreign legion, Lebanon and Afghanistan deployment Treatment- resistant and combat-related PTSD	Total participants: *N* = 3, drop-out: n.r., age: n.r., gender: 3 males	3MDR	Measurements: n.r. Effect size: n.a. Summary: Positive treatment results for three patients with treatment-resistant and combat-related PTSD. Before 3MDR the patients were treated with guidelines trauma focused interventions for 3 months to 6 years without symptom relief
Van Gelderen et al. (2020)	Netherlands	DSM-5	CAPS-5	RCT	Veterans with treatment-resistant and combat-related PTSD Treatment-resistance were defined as persisting PTSD diagnosis and lack of improvement in PTSD symptoms following a trauma-focused therapy with a treatment duration of at least 6 months	Total participants: *N* = 43, drop-out: 3 (7%), mean age: 42.1, gender: 42 (97%) males VR treatment group (3MDR): *n* = 22, drop-out: 2 (9%), mean age: 42.4, gender: 21 (95%) males Active control group (non-specific treatment component control group, NTCC): *n* = 21, drop-out: 1 (4.7%), mean age: 41.9, gender: 21 (100%) males	3MDR (after 6 sessions 3MDR patients were allowed to receive other treatments to process any therapeutic material, mean amount of treatment weeks = 10.5) vs. NTCC (non-trauma- focused treatment e.g., case management, stabilizing interventions, psychoeducation etc. up to 16 weeks; mean amount of treatment weeks = 8.74)	Measurements: Pre, 6, 12, and 16 weeks after randomization Effect size (CAPS-5): Hedges' *g*_6weeks_ = n.r. and n.a.^#^ Cohen's *d*_12weeks_ = n.r. and n.a.^#^ Cohen's *d*_16weeks_ = 0.83 (favoring 3MDR) Summary: Results showed that 3MDR reduces PTSD symptoms in Veterans with treatment-resistant and combat-related PTSD. However, no significant differences were found for secondary outcomes measures (e.g., PCL-5) and no long-term effects were assessed. The authors conclude that phase III trials with larger sample sizes are warranted
Vermetten et al. (2013)	Netherlands	n.r.	PCL-5	Case study	War veterans with treatment-resistant and combat-related PTSD.	Total participants: *N* = 2, drop-out: n.r., age: n.r., gender: n.r.	3MDR	Measurements: n.r. Effect size: n.a. Summary: Preliminary results of two cases suggest that 3MDR is perhaps a treatment for Treatment-resistant and combat-related PTSD
Kengne et al. (2018)	Canada	n.r.	n.r.	Case study	Civilian truck driver, who was suffering from PTSD following an accident.	Participant: *N* = 1, drop-out: n.r., age: n.r., gender: 1 male	ACET	Measurements: n.r. Effect size: n.a. Summary: PTSD related outcome measures were not reported. However, the authors see this single case study as “the first step for the validation” (p.8) of ACET and more studies are needed to test the efficacy
Menelas et al. (2018)	Canada	n.r.	PCL-5	Case study	Two civilian truck drivers, who were suffering from PTSD following an accident.	Total participants: *N* = 2, drop-out: n.r., age: 50 and 36 years old, gender: 2 (100%) males	ACET	Measurements: n.r. Effect size: n.r. Summary: PCL-5 decreases were found for PTSD following a truck accident. Authors highlighted that Patient b was “willing to try a road test with a monitor” (p. 11) after ACET was completed. Further studies are needed to replicate and generalize these results

*3MDR, multi-modular motion-assisted memory desensitization and reconsolidation; ACET, action-centered exposure therapy; CAPS, clinician-administered PTSD scale, measured via CAPS total sum score; CAPS-5, clinician-administered PTSD scale for DSM-5, measured via CAPS-5 total sum score; DCS, D-cycloserine; DSM-5, Diagnostic and Statistical Manual of Mental Disorders 5th Edition; DSM-IV, Diagnostic and Statistical Manual of Mental Disorders 4th Edition; DSM-IV-TR, Diagnostic and Statistical Manual of Mental Disorders 4th Edition Text Revision; Hedges' g^#^, a Hedges'g marked by “#” was recalculated. For the recalculation, we used means, standard deviations and sample sizes for the respective studies and followed the procedure by Hedges and Olkin (2014); min, minutes; MST, military sexual trauma; n.a., not applicable; n.a.^#^, not applicable, because these studies did not reported standard deviations. Instead they reported mean values and 95% confidence intervals; n.r., not reported; NTCC, non-specific treatment component control group; PCL-5, PTSD checklist for DSM-5, measured via PCL-5 total sum score; PE, prolonged exposure; post, post-treatment assessment; Pre, pre-treatment assessment; PTSD, post-traumatic stress disorder; RCT, randomized controlled trial; tDCS, transcranial direct current stimulation, patients in the tDCS group received additionally to VRET 25 minutes of two milliampere electrostimulation; TMT, trauma management therapy (Turner et al., 2005); VRET, virtual reality exposure therapy; WTC, World Trade Center*.

Regarding the main research question, the results indicate that there are three different approaches to a virtual trauma intervention, namely, VRET (Difede et al., 2013; Arens, 2014; Rothbaum et al., 2014; Reger et al., 2016; Beidel et al., 2017a,b; McLay et al., 2017; Loucks et al., 2018; Maples-Keller et al., 2018; Van‘t Wout et al., 2018), 3MDR (Vermetten et al., 2013; Jetly et al., 2017; Nijdam and Vermetten, 2018; Van Gelderen et al., 2018, 2020; Bisson et al., 2020), and action-centered exposure therapy (ACET) (Kengne et al., 2018; Menelas et al., 2018). The following describes the results of all research questions separated based on the application of VRET, 3MDR, and ACET (see Table 2 for the results of the qualitative analysis for each study and Table 3 for an overview of the results).

**Table 2:** Results of the qualitative analysis for each study.

	**Research question 1**			**Research question 2**		**Research question 3**	**Research question 4**
**References**	**Therapeutic framework**	**Transfer to VR**	**Manualized**	**Hardware**	**Software**	**Social and spatial presence**	**Efficacy for PTSD patients with imaginary problems**
**Virtual reality exposure therapy (VRET)**							
Arens (2014)	PE	Virtual re-creation of the patient's traumatic recounting	Number of sessions and period of time: 29, 90 min therapy sessions over a period of 3 weeks Virtual exposure sessions: Nine exposure sessions with ca. 45–80 min of virtual exposure Virtual exposure was conducted until within session habituation (50% reduction of the SUD) was achieved Therefore, no specific time limit of virtual exposure was set Medication: No Homework: Yes, *in vivo* excersies were included Furthermore, if between session habituation (anxiety did not increase during a virtual exposure session) was achieved, the therapist switched to *in vivo* exposure	HMD (eMaginZ800)	Virtual Iraq/Afghanistan	Was not measured	Was not evaluated
Beidel et al. (2017a)	PE	Virtual re-creation of the patient's traumatic recounting	Number of sessions and period of time: 29, 90–120 min therapy sessions over a period of 17 weeks Virtual exposure sessions: 14 exposure sessions with ca. 20–100 min of virtual exposure Virtual exposure was conducted until within session habituation (50% reduction of the SUD) was achieved Therefore, no specific time limit of virtual exposure was set Medication: No Homework: Yes, *in vivo* excersies were included Furthermore, if between session habituation (anxiety did not increase during a virtual exposure session) was achieved, the therapist switched to *in vivo* exposure	HMD (HMZ-T3W)	BRAVEMIND	Was not measured	Was not evaluated
Beidel et al. (2017b)	PE	Virtual re-creation of the patient's traumatic recounting	Number of sessions and period of time: 29, 90–120 min therapy sessions over a period of three weeks Virtual exposure sessions: 14 exposure sessions with ca. 20–100 min of virtual exposure Virtual exposure was conducted until within session habituation (50% reduction of the SUD) was achieved Therefore, no specific time limit of virtual exposure was set Medication: No Homework: Yes, *in vivo* excersies were included Furthermore, if between session habituation (anxiety did not increase during a virtual exposure session) was achieved, the therapist switched to *in vivo* exposure	HMD (eMaginZ800)	Virtual Iraq/Afghanistan	Was not measured	Was not evaluated
Difede et al. (2013)	PE	Virtual re-creation of the patient's traumatic recounting	Number of sessions and period of time: Twelve, 90 min therapy sessions over a period of 12 weeks Virtual exposure sessions: Ten exposure sessions with ca. 45 min of virtual exposure Referring to Difede et al. (2007) the virtual exposure was also conducted until within-session habituation was attained (50% reduction of SUD) Medication: Yes, patients took D-cycloserin (100 mg) or placebo 90 min before the exposure session Homework: Yes, *in vivo* excersies were included	HMD (Kaiser XL-50)	Virtual version of the WTC attacks	Was not measured	Was not evaluated
Loucks et al. (2018)	PE	Virtual re-creation of the context and settings factors. For MST it is not attempt to recreate a sexual assault.	Number of sessions and period of time: Six to 12, 90 min therapy sessions over a period of 6 to 9 weeks Virtual exposure sessions: Four to 10 exposure sessions with ca. 45 min of virtual exposure Did n.r. whether within-session habituation in sense of 50% reduction of SUD was attained Medication: No Homework: Yes, *in vivo* excersies were included	HMD (eMaginZ800)	BRAVEMIND	Was not measured	Was not evaluated
Maples-Keller et al. (2018)	PE	Virtual re-creation of the patient's traumatic recounting	Number of sessions and period of time: Seven to twelve, 90 min therapy sessions over a period of 7 to 12 weeks Virtual exposure sessions: Six to 11 exposure sessions with ca. 30–45 min of virtual exposure Did n.r. whether within-session habituation in sense of 50% reduction of SUD was attained Medication: Yes, patients took dexamethasone (0.5 mg) or placebo the night before virtual exposure Homework: No	HMD (eMaginZ800)	Virtual Iraq/Afghanistan	Was not measured	Was not evaluated
McLay et al. (2017)	PE	Virtual re-creation of the patient's traumatic recounting	Number of sessions and period of time: Eight to 12, 90 min therapy sessions over a period of 9 weeks Virtual exposure sessions: Five to nine exposure sessions with 30–45 min of virtual exposure Did n.r. whether within-session habituation in sense of 50% reduction of SUD was attained Medication: No Homework: Yes, *in vivo* excersies were included	HMD (eMaginZ800)	Virtual Iraq/Afghanistan	Was not measured	Was not evaluated
Reger et al. (2016)	PE	Virtual re-creation of the patient's traumatic recounting	Number of sessions and period of time: Ten, 90–120 min therapy sessions over a period of 5 to 10 weeks Virtual exposure sessions: Eight exposure sessions with ca. 45 min of virtual exposure Did n.r. whether within-session habituation in sense of 50% reduction of SUD was attained Medication: No Homework: Yes, *in vivo* excersies were included	HMD (eMaginZ800)	Virtual Iraq/Afghanistan	Was not measured	Was not evaluated
Rothbaum et al. (2014)	PE	Virtual re-creation of the patient's traumatic recounting	Number of sessions and period of time: Six, 90 min therapy sessions over a period of 6 weeks Virtual exposure sessions: Five exposure sessions with 45 min of virtual exposure Medication: Yes, patients took D-cycloserine (50 mg), alprazolam (0.25 mg) or the placebo medication 30 min before exposure Did n.r. whether within-session habituation in sense of 50% reduction of SUD was attained Homework: No	HMD (eMaginZ800)	Virtual Iraq/Afghanistan	Was not measured	Was not evaluated
Van‘t Wout et al. (2018)	PE	Virtual re-creation of the patient's traumatic recounting	Number of sessions and period of time: Six, 90 min therapy sessions over a period of 2 weeks Virtual exposure sessions: Six exposure sessions with 30–45 min of virtual exposure Medication: Yes, during virtual exposure patients received tDCS or sham tDCS Did n.r. whether within-session habituation in sense of 50% reduction of SUD was attained Homework: No	HMD (eMaginZ800)	Virtual Iraq/Afghanistan	Was not measured	Was not evaluated
**Multi-modular motion-assisted memory desensitization and reconsolidation (3MDR)**							
Bisson et al. (2020)	EMDR	Patients walk toward individualized trauma-related symbolic images in a CAVE	Number of sessions and period of time: Nine, 60 min therapy sessions over a period of 9 weeks Virtual exposure sessions: Six virtual exposure sessions with ca. 45 min of virtual exposure Did n.r. whether within-session habituation in sense of 50% reduction of SUD was attained Medication: No Homework: No	CAVE (GRAIL)	Individualized trauma-associated images	Was not measured	Was not evaluated. However, only treatment-resistant PTSD patients were included in this study
Jetly et al. (2017)	EMDR	Patients walk toward individualized trauma-related symbolic images in a CAVE	Number of sessions and period of time: Nine, ca. 40 min therapy sessions over a period of 8 weeks Virtual exposure sessions: Six virtual exposure sessions with ca. 30 min of virtual exposure Did n.r. whether within-session habituation in sense of 50% reduction of SUD was attained Medication: No Homework: No	CAVE (CAREN)	Individualized trauma-associated images	Was not measured	Was not evaluated. However, only treatment-resistant PTSD patients were included in this study
Nijdam and Vermetten (2018)	EMDR	Patients walk toward individualized trauma-related symbolic images in a CAVE	Number of sessions and period of time: Was not reported Virtual exposure sessions: Six virtual exposure sessions (duration of virtual exposure was not reported) Did n.r. whether within-session habituation in sense of 50% reduction of SUD was attained Medication: No Homework: No	CAVE (CAREN)	Individualized trauma-associated images	Was not measured	Was not evaluated. However, only treatment-resistant PTSD patients were included in this study
Van Gelderen et al. (2018)	EMDR	Patients walk toward individualized trauma-related symbolic images in a CAVE	Number of sessions and period of time: Was not reported Virtual exposure sessions: Was not reported Did n.r. whether within-session habituation in sense of 50% reduction of SUD was attained Medication: No Homework: No	CAVE (CAREN)	Individualized trauma-associated images	Was not measured	Was not evaluated. However, only treatment-resistant PTSD patients were included in this study
Van Gelderen et al. (2020)	EMDR	Patients walk toward individualized trauma-related symbolic images in a CAVE	Number of sessions and period of time: Six + 10 optional, 70–90 min therapy session over a period of 6–16 weeks Virtual exposure sessions: Six virtual exposure sessions with 30–45 min of virtual exposure Did n.r. whether within-session habituation in sense of 50% reduction of SUD was attained Medication: No Homework: No	CAVE (CAREN)	Individualized trauma-associated images	Was not measured	Was not evaluated. However, only treatment-resistant PTSD patients were included in this study
Vermetten et al. (2013)	EMDR	Patients walk toward individualized trauma-related symbolic images in a CAVE	Number of sessions and period of time: Six, 45 min therapy session over a period of 4 weeks Virtual exposure sessions: Four virtual exposure sessions (duration of virtual exposure was n.r.) Did n.r. whether within-session habituation in sense of 50% reduction of SUD was attained Medication: No Homework: No	CAVE (CAREN)	Individualized trauma-associated images	Was not measured	Was not evaluated. However, only treatment-resistant PTSD patients were included in this study
**Action-centered exposure therapy (ACET)**							
Kengne et al. (2018)	Inhibitory learning	Active interaction with a virtual trauma-associated environment	Number of sessions and period of time: Eight therapy session (session duration and period of weeks were n.r.) Virtual exposure sessions: Six virtual exposure sessions (duration of virtual exposure was n.r.) Did n.r. whether within-session habituation in sense of 50% reduction of SUD was attained Medication: No Homework: No	HMD (HMZ-T2)	Self-programmed virtual country roads, highways, and cities	Was not measured	Was not evaluated
Menelas et al. (2018)	Inhibitory learning	Active interaction with a virtual trauma-associated environment	Number of sessions and period of time: Eight therapy session over a period of 4 weeks Virtual exposure sessions: Six virtual exposure sessions (duration of virtual exposure was not reported) Did n.r. whether within-session habituation in sense of 50% reduction of SUD was attained Medication: No Homework: No	HMD (HMZ-T2)	Self-programmed virtual country roads, highways, and cities	Was not measured	Was not evaluated

*3MDR, multi-modular motion-assisted memory desensitization and reconsolidation; ACET, action-centered exposure therapy; CAREN, computer-assisted rehabilitation environment; CAVE, cave automatic virtual environment; EMDR, eye movement desensitization and reprocessing; GRAIL, gait real-time analysis interactive lab; HMD, head-mounted display; mg, milligram; min, minute; MST, military sexual trauma; n.r., not reported; PE, prolonged exposure; PTSD, post-traumatic stress disorder; SUD, subjective units of distress; tDCS, transcranial direct current stimulation; VR, virtual reality; VRET, virtual reality exposure therapy; WTC, World Trade Center*.

**Table 3:** Overview of results.

	**VRET**	**3MDR**	**ACET**
**General overview**			
Included studies	−7 RCTs (patients: *N =* 555) - 2 Pilot studies (patients: *N =* 127) - 1 Case study (patient: *N =* 1)	−2 RCTs (patients: *N =* 85) - 1 Pilot study (patients: *N =* 8) - 3 Case studies (patients: *N =* 6)	−2 Case studies (patients: *N =* 2)
Country	- USA: 10/10 studies	- Canada: 1/6 study - Netherlands: 4/6 studies - UK: 1/6 study	- Canada: 2/2 studies
Trauma type	−8/10 war veterans with combat-related PTSD - 1/10 war veterans with PTSD after MST - 1/10 civilians with PTSD after a terrorist attack (WTC)	−6/6 war veterans with combat-related and treatment-resistant PTSD	−2/2 civilian truck drivers with PTSD after a truck driver accident
**Research questions**			
1.1) Therapeutic framework ( 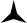 )	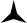 PE	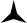 EMDR	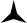 Inhibitory learning
1.2) How it was transferred to VR (summary) ( 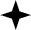 )	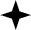 Summary: Virtual re-creation of the patient's traumatic recounting during virtual exposure	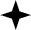 Summary: Patients walk toward individualized trauma-related symbolic images, which increased simultaneously and continuously in size. After each image patients perform virtual dual-attention tasks to stimulate the patient's working memory and facilitate reconsolidation	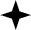 Summary: Active interaction with a virtual trauma-associated environment during virtual exposure
1.3) Manualized (★)			
1.3.1) Number of sessions and period of time	★7/10 studies used six to 12, ca. 90 min therapy sessions over a period of 6 to 12 weeks ★3/10 studies used 29, 90–120 min therapy sessions over a period of 3–17 weeks	★1/6 used six, 45 min therapy sessions over a period of 4 weeks ★2/6 studies used nine, 40–60 min therapy sessions over a period of 8 to 9 weeks ★1/6 used nine plus 10 optional 70–90 min therapy sessions over a period of 6–16 weeks ★2/6 did n.r. the number and time of therapy sessions	★2/2 used eight therapy sessions ★1/2 reported a period of 4 weeks and the other one did n.r. the period of weeks ★No study reported the exact time of therapy sessions
1.3.2) Virtual exposure sessions	★7/10 studies used six to 11 virtual exposure sessions with ca. 30–45 min of virtual exposure ★3/10 studies used nine to 14 virtual exposure sessions with ca. 20–100 min of virtual exposure ★4/10 studies explicitly reported that virtual exposure was conducted until within-session habituation was attained	★5/6 studies used six virtual exposure sessions, with ca. 30–45 min of virtual exposure ★1/6 n.r. the number and time of virtual exposure sessions ★0/6 studies did n.r. whether virtual exposure was conducted until within-session habituation was attained	★2/2 studies used six virtual exposure sessions ★The duration of virtual exposure was n.r. ★0/2 studies did n.r. whether virtual exposure was conducted until within-session habituation was attained
1.3.3) Medication	★4/10 studies used medication (Alp., DCS, DMT, tDCS)	★6/6 did not use medication	★2/2 did not use medication
1.3.4) Homework (*in vivo* exercise)	★7/10 studies included *in vivo* exercises between the therapy sessions (e.g., visiting crowded places, sitting with one's back to a doorway)	★6/6 studies did not include *in vivo* exercise	★2/2 studies did not include *in vivo* exercise
2.1) Hardware (❖)	❖ 8/10 studies used eMaginZ800 (HMD, released in 2005) ❖ 1/10 study used ProView XL-50 (HMD, released in 2006) ❖ 1/10 study used HMZ-T3W (HMD, released in 2013)	❖ 5/6 studies used CAREN (computer-assisted rehabilitation environment, first release in 2000, assignable to a CAVE) ❖ 1/6 study used GRAIL (Gait Real-time Analysis Interactive Lab, assignable to a CAVE)	❖ HMZ-T2 (HMD, released in 2012) ❖ Logitech G27 3 Driving Force GTracing wheel (wheel, gas, and brake pedal)
2.2) Software (♦)	♦ 7/10 studies used the virtual Iraq / Afghanistan ♦ 2/10 studies used BRAVEMIND ♦ 1/10 study used a virtual version of the World Trade Center attacks	♦ Patients brought five to seven trauma-associated images with them, which were subsequently projected on the curved screen	♦ Multiple country roads, highways, and cities were used as virtual environment (self-programmed)
3) Social and spatial presence (•)	•Was not measured	•Was not measured	•Was not measured
4) Reaches PTSD patients with imaginary problems (■)	■ Was not evaluated	■ Was not evaluated ■ However, two recent RCTs showed significant symptom decrease for treatment-resistant PTSD	■ Was not evaluated

*3MDR, multi-modular motion-assisted memory desensitization and reconsolidation; ACET, action-centered exposure therapy; Alp., Alprazolam; CAVE, cave automatic virtual environment; EMDR, eye movement desensitization and reprocessing; DCS, D-cycloserine; DMT, Dexamethasone; HMD, head-mounted display; IED, improvised explosive device; min, minute; MST, military sexual trauma; n.r., not reported; PE, prolonged exposure; PTSD, post-traumatic stress disorder; RCTs, randomized controlled trials; tDCS, transcranial direct current stimulation; UK, United Kingdom; USA, United States of America; VR, virtual reality; VRET, virtual reality exposure therapy; WTC, World Trade Center*.

### Virtual Reality Exposure Therapy (VRET)

#### Therapeutic Framework Transferred Into VRET

VRET is based on the therapeutic framework of PE. Here, a patient's traumatic experience was administered virtually, and not through an imagination-based approach (Rothbaum et al., 2010; Leaman et al., 2013). Usually, the patients recount their traumatic memories during the VRET study's virtual confrontations and the therapists match the virtual environments to the recounted experiences (Rothbaum et al., 2010). This type of regeneration/virtual re-creation was presumed to enhance the patient access to trauma-associated stimuli and memories (Rothbaum et al., 2010; Leaman et al., 2013). Nine of 10 (90%) studies included soldiers or veterans with combat-related PTSD, with one specializing on PTSD after an MST (Loucks et al., 2018). One (10%) study examined civilians with PTSD related to the attack on the WTC (Difede et al., 2013).

In 90% of VRET studies, virtual Iraq/Afghanistan or an updated version, called Bravemind, has been used (Rizzo et al., 2005, 2017). These virtual environments include comprehensive prototype scenarios of combat-related PTSD experiences, such as, riding in a Humvee through a desert (Rizzo et al., 2005). Moreover, a clinical interface was integrated into virtual Iraq/Afghanistan and the Bravemind systems (Rizzo et al., 2017), which enabled the therapist to customize the virtual environments in real-time (e.g., daytime duration, weather conditions, and ambient sounds), to match the patient recounted experiences (Leaman et al., 2013; Rizzo et al., 2017). The clinical interface also allows the therapist to add trigger stimuli, such as explosions or gunfire attacks (Rizzo et al., 2005). Typically, this feature was used in later sessions, when the therapist and patient focus on trauma hotspots, i.e., the portions of traumatic memories causing high levels of anxiety and emotional distress (Leaman et al., 2013).

Several treatment protocols for VRET exist (McLay et al., 2011; Leaman et al., 2013; Rothbaum et al., 2014; Reger et al., 2016). Therefore, the actual procedure varied across studies. The treatment protocol began with one to two preparatory sessions in 70% of the VRET studies (Difede et al., 2013,^3^; Rothbaum et al., 2014; Reger et al., 2016; McLay et al., 2017; Loucks et al., 2018; Maples-Keller et al., 2018; Van‘t Wout et al., 2018). Here, the clinician provided an overview of VRET, discussed the treatment duration and adherence, gathered the patient traumatic experience-related information, and presented the rationale of *in vivo* and virtual exposure. These sessions were followed by five (Rothbaum et al., 2014) to 10 (McLay et al., 2017) 90-min sessions, including 30–45 min for virtual exposure and an approximately 20-min conversation to support patients in processing their trauma-related notions, thoughts, and feelings.

In contrast, three studies conducted virtual exposure until the patients achieved within-session habituation (Arens, 2014; Beidel et al., 2017a,b). Within-session habituation was operationalized with a 50% lower anxiety than the session peak. The session peak was measured using a subjective unit of distress (SUD) index, on a scale from 0 to 8 (Arens, 2014; Beidel et al., 2017a,b). Therefore, the first virtual confrontation sessions continued for 90–120 min and the later ones for 15–20 min (Arens, 2014; Beidel et al., 2017a,b). Furthermore, if the patients' distress level did not increase during virtual exposure (between session habituations), the therapists switched to *in vivo* exposure. Therefore, the therapists used actual places and situations related to the patient's traumatic experience (e.g., crowded places, driving on roads similar to the location of an IED explosion) (Arens, 2014; Beidel et al., 2017a,b).

Seven studies included at-home *in vivo* exposure exercises to avoided situations (e.g., sleeping with a bathroom door open or sitting with one's back to a doorway) (Difede et al., 2013; Arens, 2014; Reger et al., 2016; Beidel et al., 2017a,b; McLay et al., 2017; Loucks et al., 2018), while three did not (Rothbaum et al., 2014; Maples-Keller et al., 2018; Van‘t Wout et al., 2018). In summary, the therapists customized the virtual environment to the patient recounting during the virtual exposure in VRET studies. However, due to the heterogeneity in treatment protocols, the generalization of VRET procedures is difficult.

#### Hardware and Software Used for VRET

Regarding the second research question, in 80% of VRET studies (Arens, 2014; Rothbaum et al., 2014; Reger et al., 2016; Beidel et al., 2017a,b; McLay et al., 2017; Loucks et al., 2018; Maples-Keller et al., 2018; Van‘t Wout et al., 2018), *eMaginZ800* HMD (released in 2005), which has a pixel resolution of 800 × 600 and a 40° diagonal field of view for each eye (Rizzo et al., 2005), was used. Difede et al. (2013) applied a ProView XL-50 HMD (released in 2006), which has a pixel resolution of 1,042 × 768 with a 40° horizontal field of view. Beidel et al. (2017a) applied an HMZ-T3W HMD (released in 2013), which has a pixel resolution of 1,280 × 720 with a 45° horizontal field of view.

Most of the studies (90%) used the virtual Iraq/Afghanistan developed by Rizzo et al. (2005) or the updated version Bravemind (Rizzo et al., 2017) as a virtual trauma environment. Bravemind is based on the first version of Virtual Iraq/Afghanistan (Rizzo et al., 2005) and was updated and further developed in 2011 using the Unity 3d game engine (Rizzo and Shilling, 2017; Rizzo et al., 2017). The four original scenarios were rebuilt using the Unity 3d engine, and 10 additional preprogrammed situations were added (e.g., a rural Afghan village and a roadway checkpoint). In both virtual environments, the patients control their perspectives through head movements and navigate through the virtual environment using a gaming controller.

Difede et al. (2013) used a virtual version of the World Trade Center (WTC) attack as a virtual environment that consists of 13 preprogrammed scenarios. Based on the idea of a hierarchical exposure, these 13 preprogrammed scenarios vary in the intensity of the presented material. The first scene starts with a jet flying over the WTC towers without a crash, hit, or explosion. The following scenario shows a jet crashing into the first tower, but the tower does not collapse. The last scene shows the jets crash into both towers. The towers collapse, dust clouds appear, and human screaming can be heard.

#### Spatial and Social Presence in VRET

The patients' spatial or social presence during virtual trauma exposure was not measured in any of the 10 studies.

#### VRET for PTSD Patients With Imaginary Problems

Whether VRET is particularly effective for patients with PTSD and imagination difficulties has not been examined in any of the aforementioned studies.

#### Additional Information (Study Objectives and Efficacy)

A total of 10 (55.5%) VRET studies were included in the present scoping review, with seven being RCTs (Difede et al., 2013; Rothbaum et al., 2014; Reger et al., 2016; Beidel et al., 2017a; McLay et al., 2017; Maples-Keller et al., 2018; Van‘t Wout et al., 2018), two pilots (Beidel et al., 2017b; Loucks et al., 2018) and one single-case study (Arens, 2014).

The studies' objectives were quite heterogeneous. Three out of 10 studies explored whether the efficacy of VRET may be increased through additional medication (Difede et al., 2013; Rothbaum et al., 2014; Maples-Keller et al., 2018). Maples-Keller et al. (2018) examined whether the administration of dexamethasone improved the efficacy of VRET compared to placebo treatment. Rothbaum et al. (2014) analyzed to what extent D-cycloserine and alprazolam influenced the efficacy of VRET, also in comparison to a placebo group. Furthermore, Difede et al. (2013) examined whether D-cycloserine improved the efficacy of VRET in comparison to a placebo group. None of these studies revealed a significant difference at the post time measurement compared to the placebo group (Difede et al., 2013; Rothbaum et al., 2014; Maples-Keller et al., 2018). All studies used the Clinician-Administered PTSD Scale (CAPS; Blake et al., 1995) as the primary outcome measure.

Further three out of 10 studies examined whether VRET may be integrated into Trauma Management Therapy (TMT; Turner et al., 2005). Three studies with different designs showed that TMT in combination with VRET led to a clinically relevant and significant reduction of PTSD symptoms from pre- to post and 6-month-follow-up (Arens, 2014; Beidel et al., 2017a,b). Beidel et al. (2017a) compared whether the combination of TMT with VRET is more effective than psychoeducation modules with VRET. They did not reveal any significant differences between the conditions at post assessment and at the 6-month-follow-up (Beidel et al., 2017a).

Two feasibility studies examined whether VRET can be used to treat MST (Loucks et al., 2018) and whether transcranial direct current stimulation (tDCS; Nitsche et al., 2008) increases the efficacy of VRET (Van‘t Wout et al., 2018). These studies showed that VRET reduced the PTSD symptoms after MST significantly from pre- to post and 3-month-follow-up (Loucks et al., 2018), and that tDCS could improve the efficacy of VRET (Van‘t Wout et al., 2018).

Reger et al. (2016) conducted an RCT to examine whether there was a significant difference between the efficacy of VRET, PE and a minimal attention waitlist. They showed that VRET as well as PE were superior to the control group (Reger et al., 2016). Contrary to expectation, there was no significant difference between VRET and PE at post-treatment, whereas VRET was significantly inferior to PE after a 3-month-follow-up (Reger et al., 2016).

McLay et al. (2017) also conducted an RCT to examine whether the efficacy differed between immersive (HMD) and non-immersive (PC) VRET. McLay et al. (2017) found no significant difference in CAPS scores between the conditions at the post-treatment and 3-month-follow-up.

### Multi-Modular Motion-Assisted Memory Desensitization and Reconsolidation (3MDR)

#### Therapeutic Framework Transferred Into 3MDR

The 3MDR framework is a model for the treatment of PTSD, focusing on therapy-resistant PTSD (trPTSD; Koek et al., 2016), where the mechanisms of EMDR, multi-sensory input, walking on a treadmill, and dual attention tasks are combined (Vermetten et al., 2013; Van Gelderen et al., 2018, 2020). Van Gelderen et al. (2018) hypothesized that avoidance behavior of trPTSD is particularly pronounced, making access to traumatic memories more difficult. Therefore, new therapeutic approaches are needed to reduce avoidance behavior, increase therapy adherence, and reduce symptoms of trPTSD (Vermetten et al., 2013; Nijdam and Vermetten, 2018; Van Gelderen et al., 2018, 2020). All studies included war veterans with combat-related and treatment-resistant PTSD.

In addition, all studies used the same 3MDR manualized treatment protocol, which posits 6 weekly 90 min sessions (Van Gelderen et al., 2020). Each session includes a preliminary briefing (20 min), a virtual trauma confrontation on a treadmill (for approximately 50 min), and a review discussion (20 min). Before the virtual exposure, one or two preparatory sessions are conducted (45 min). Here, the goal is to inform the patient about the procedure, offer psychoeducation, and select photographs and music that will be integrated into the virtual trauma confrontation (Van Gelderen et al., 2020).

One central part of the preparatory sessions is the selection process of 10–20 highly affective photographs associated with the traumatic experience (Bisson et al., 2020; Van Gelderen et al., 2020). The patients can choose any photograph they want to see (Bisson et al., 2020). For instance, they can choose their own photographs or select images from the Internet (e.g., landscapes or an area on Google Maps) (Van Gelderen et al., 2020). The selected images are then arranged according to the distress they evoke. Therefore, each photograph will be rated with a SUD using a score of 0–10. For each session, a maximum of seven pictures were selected and arranged according to their SUD scores (Bisson et al., 2020). The chosen images will be projected on the CAVE and enable the creation of an idiosyncrasy virtual environment rather than a generic one (Van Gelderen et al., 2020). The virtual confrontation will start with the least emotionally image (with the lowest SUD) and will finish with the highest. This procedure can be repeated throughout the sessions, and it is also possible to reorder or reselect the images for each session (Van Gelderen et al., 2020).

As another precondition, the patient selects two different pieces of music (e.g., songs or natural sounds) (Bisson et al., 2020). The first is for a warm-up and the second is for a cool-down walk. The warm-up music aims to evoke associations related to the trauma-related period and to bring the patient back to the time of the trauma (Bisson et al., 2020; Van Gelderen et al., 2020). The cool-down music helps the patient return to the here and now and should not be trauma related.

After the preparatory sessions and preliminary discussion, the virtual trauma confrontation starts with a technical briefing (Van Gelderen et al., 2020). Therefore, the operator, who is in the room for the entire duration of the virtual exposure, tethers the patient in the safety harness, provides information regarding safety, and adjusts the speed of the treadmill. The introductory phase then begins, which consists of the warm-up music and an initial walk until the patient reaches the first virtual tunnel (Bisson et al., 2020; Van Gelderen et al., 2020).

The patients enter the first tunnel and by doing so move toward the first selected image, which increases simultaneously and continuously in size (Van Gelderen et al., 2020). While moving forward, the patient should describe the photograph and the associative memories. The patients should then talk about their feelings evoked by the image at that moment (now feelings). When the patients verbalize their current feelings, the therapist repeats them, the operator types them in, and they appear in real-time on the screen (Van Gelderen et al., 2020).

After this reprocessing of trauma-related memories and feelings, the reconsolidation process starts with a dual-attention task (Van Gelderen et al., 2018). Therefore, a red ball appears on the screen. This red ball moves from left to right with different numbers. The patients' task is to call out these numbers and simultaneously concentrate on the memories and feelings they just named on the screen. The patients should call out six or more numbers. If they are unable to do so, the task is extended by the operator (Van Gelderen et al., 2020).

This sequence will be repeated until the patient completes the last (7th) image (Bisson et al., 2020). The outro sequence then begins. The patients' cool-down music plays, and the treadmill begins to slow down and finally stops. The operator helps the patient out of the harness, and the virtual trauma confrontation ends with a brief discussion between the patient, operator, and therapist (Van Gelderen et al., 2020).

#### Hardware and Software Used for 3MDR

In five out six studies, the computer-assisted rehabilitation environment (CAREN) was used as an immersive technology. CAREN, released in 2000, consists of a projection surface 6-m long and curved 180°, with a 5.1 Dolby Surround audio system, a tracking system, and an omnidirectional treadmill (Mert et al., 2013; Vermetten et al., 2013; Jetly et al., 2017; Van Gelderen et al., 2018, 2020). Bisson et al. (2020) used the gait real-time analysis interactive lab (GRAIL), which consists of a 180° projection screen with four projectors, a surround and motion-capture system, and an instrumented dual-belt treadmill (Bisson et al., 2020).

In all studies the patients brought their own trauma-associated images with them, which were subsequently projected on the curved screen (Vermetten et al., 2013; Jetly et al., 2017; Nijdam and Vermetten, 2018; Van Gelderen et al., 2018, 2020; Bisson et al., 2020). These 2D images were used as the virtual environment in all 3MDR studies.

#### Spatial and Social Presence in 3MDR

The patients' spatial or social presence was not measured in any of the six studies.

#### 3MDR for PTSD Patients With Imaginary Problems

Whether 3MDR is particularly effective for patients with PTSD and imagination difficulties was not evaluated in any of the studies. However, all 3MDR studies were conducted with a focus on veterans or soldiers with treatment-resistant PTSD. Treatment resistance was defined as a persisting PTSD diagnosis following trauma-focused therapy. Van Gelderen et al. (2020) reported that the patients had, on average, four unsuccessful PTSD treatments. The sample by Bisson et al. (2020) also consisted of patients who had tried at least one trauma-focused psychological treatment (EMDR or trauma-focused cognitive behavioral therapy).

#### Additional Information (Study Objectives and Efficacy)

A total of six (33.3%) 3MDR studies were included in the present scoping review, with two being RCTs (Bisson et al., 2020; Van Gelderen et al., 2020), one pilot (Jetly et al., 2017), and three single-case studies (Vermetten et al., 2013; Nijdam and Vermetten, 2018; Van Gelderen et al., 2018). The objectives of the studies were considerably homogenous. Whether 3MDR is an efficient treatment tool for treatment-resistant PTSD was examined in all the 3MDR studies.

Bisson et al. (2020) conducted an RCT of British military servicemen with trPTSD to examine whether 3MDR was superior to the waitlist group. Patients in the waitlist group received 3MDR after a delay of 6 weeks (Bisson et al., 2020). As expected, 3MDR was significantly superior to the waitlist at post-treatment (*d* = 0.65), and there was no significant difference between the groups after 26 weeks (at this time, both groups received 3MDR) (Bisson et al., 2020).

Van Gelderen et al. (2020) also conducted an RCT of Dutch veterans with trPTSD to examine whether 3MDR is superior to an active control group. Patients in the active control group received non-trauma-focused treatments such as stabilizing intervention or cognitive behavioral therapy without exposure or cognitive restructuring of trauma-related cognitions for up to 16 weeks. Patients in the 3MDR group received six standardized 3MDR sessions. In addition, there was the possibility of 10 optional sessions, depending on patients' need or the indications of the therapists. As expected, 3MDR was significantly superior to the active control group after 16 weeks of treatment (*d* = 0.83). However, immediately after the six 3MDR sessions (after week 6), no significant differences were shown.

The other pilot and single-case studies also revealed clinically relevant symptom reductions for trPTSD (Vermetten et al., 2013; Jetly et al., 2017; Nijdam and Vermetten, 2018; Van Gelderen et al., 2018).

### Action-Centered Exposure Therapy (ACET)

#### Therapeutic Framework Transferred Into ACET

In ACET, the focus is on patients' active interaction with the virtual trauma-associated environment. Thus, the approach of inhibitory learning in exposure therapy is used. This is the main difference between ACET and VRET, which utilizes the emotional processing theory by creating virtual visualizations to supplement the patients' traumatic recounting (Foa and Kozak, 1986; Foa and McNally, 1996; Rizzo and Shilling, 2017; Kengne et al., 2018; Menelas et al., 2018). It is assumed that these virtual visualizations activate the pathological fear structure more controllably (Leaman et al., 2013). However, VRET does not focus on active interactions between the patients and virtual trauma-related environment; rather, it supplements the patients' imagination. In contrast, ACET focuses on the active interaction with the virtual trauma-associated environment, with the aim of reducing PTSD symptoms by altering the anxiety structure through new secondary inhibitory learning (Craske et al., 2014; Kengne et al., 2018; Menelas et al., 2018). In ACET, patients are encouraged to gain new experiences, for example, by accomplishing missions such as driving a truck from an industrial area to a rural one (Kengne et al., 2018). To accomplish these missions, the patients must interact with the virtual trauma-related environment (e.g., in addition to navigating and changing perspectives using head movements, it is intended that the patients explore the virtual environment independently; they can select and manipulate virtual objects and interact with artificial-intelligence-programmed road drivers) (Kengne et al., 2018; Menelas et al., 2018). Habituation is not emphasized here; rather, the focus is on the new secondary inhibitory learning, which is derived from the active interactions within the virtual environment (Craske et al., 2014; Kengne et al., 2018).

Both single-case studies were conducted with a focus on PTSD related to truck driver accidents and used the same treatment protocol, which consisted of eight sessions (Kengne et al., 2018; Menelas et al., 2018). The first two sessions were preparatory sessions. Here, the patient should become familiar with the virtual environment, learn how to interact with it, and reconnect with driving mechanisms without direct exposure to a truck (Menelas et al., 2018). Therefore, the authors programmed a flying carpet, which was controlled using a steering wheel, gas, and brake pedal, as applied in the later sessions using a truck (Kengne et al., 2018; Menelas et al., 2018). The author chose this allegory to allow the patients to reconnect with the driving mechanism without direct exposure (Kengne et al., 2018; Menelas et al., 2018).

In the third session, the patients were exposed to a truck (Kengne et al., 2018). They walked around a virtual parking lot and could choose their favorite one. In the following session they could customize the truck, for example, change the color or seat materials. In sessions four and five, the patients drove the truck, with several missions to accomplish. For instance, they were required to drive the truck to a specific destination (Kengne et al., 2018; Menelas et al., 2018). By doing so, they were exposed to different weather conditions (sun, rain, and snow) and different roads (e.g., small streets without traffic or highways with high traffic). In session six, the patients were indirectly exposed to trauma by passing a burning truck on the roadside. After this high-level exposure, sessions seven and eight focused on the driving itself with similar content as provided in sessions four and five (Kengne et al., 2018; Menelas et al., 2018).

#### Hardware and Software Used for ACET

Both single-case studies used the HMZ-T2, an HMD by Sony (released in 2012) and the head-tracking system Natural Point TrackIR 5 (Kengne et al., 2018; Menelas et al., 2018). Based on the Game Engine Unreal Engine 4, multiple country roads, highways, and cities with different weather (sun, snow, rain, etc.) and daylight conditions were programmed. Using a steering wheel, gas, and brake pedal (Logitech G27 3 Driving Force GTracing wheel), the patient steered the truck through the virtual environment as close to reality as possible (Kengne et al., 2018; Menelas et al., 2018). Furthermore, it was possible to additionally add car traffic programmed with artificial intelligence. The authors ensured that this simulated road traffic was as close to reality as possible to allow the patient to independently collect new and corrective experiences.

#### Spatial and Social Presence in ACET

The spatial or social presence was not measured in any of the single-case studies (Kengne et al., 2018; Menelas et al., 2018).

#### ACET for PTSD Patients With Imaginary Problems

Whether ACET is particularly effective for PTSD patients with imagination difficulties was not evaluated in any of the abovementioned studies (Kengne et al., 2018; Menelas et al., 2018).

#### Additional Information (Study Objectives and Efficacy)

The present scoping review identified two (11.1%) case studies on ACET (Kengne et al., 2018; Menelas et al., 2018). Both studies showed initial evidence that ACET can reduce PTSD symptoms related to truck driving accidents. The authors highlighted that, after ACET, one patient (patient_b_) was able to complete the driving training and is now working again as a truck driver (Menelas et al., 2018). However, larger replication studies are required to confirm the efficacy of ACET. Therefore, the results must be interpreted with caution (Kengne et al., 2018; Menelas et al., 2018).

## Discussion

The present scoping review revealed that, at present, research on the subject encompasses three different approaches for virtual trauma interventions, which are based on three different trauma-focused therapeutic approaches. Regarding the first research question (1), the results showed that VRET is based on PE, 3MDR is based on EMDR, and ACET is based on an inhibitory learning model. In accordance with previous reviews, this review also found that the empirical focus of previous research has been on VRET (10 out of 18 studies, with 7 out of 9 RCTs), whereas 3MDR (6 out of 18, with 2 out of 9 RCTs) and ACET (2 out of 18) represent developments that have not been considered to a large extent in previous research. However, during the next few years, it is likely that the focus of the examination will shift to 3MDR. Currently, one such study is registered in the International Standard Randomized Controlled Trial Number (ISRCTN) registry (ISRCTN11264368). Another study is registered at clinicaltrials.gov (NCT03796936), and yet another study is registered in the Netherlands Trial Register (NL6837). In contrast only a single pre-registration for VRET was found at clinicaltrials.gov (NCT0135263) and none was found for ACET^4^. Based on the three pre-registrations for 3MDR and the results of the two recent published RCTs (Bisson et al., 2020; Van Gelderen et al., 2020), we likely assume that the research focus will shift to 3MDR.

Furthermore, to answer the second part of the first research question (1) our results showed that the method of transferring the therapeutic framework into VR varies for the three types of virtual trauma interventions (see Table 3). VRET focuses on supplementing patients' traumatic recounting through virtual visualizations, which activate the pathological fear structure controllably and in conformity with the emotional processing theory (Foa and McNally, 1996; McLean and Foa, 2011). Subsequently, the affective memory can be modified through habituation and extinction experience. This process can change the pathological fear structure and reduce PTSD symptoms. For this purpose, pre-programmed virtual scenarios, which can be partly modified to aid the patient's traumatic recounting, were used (Leaman et al., 2013; Rizzo and Shilling, 2017). In 3MDR, the patients walk toward individualized trauma-related symbolic images in a CAVE, which increase continuously in size, aiming to break and minimize avoidance patterns (Van Gelderen et al., 2020). At the end of each image presentation, the patient performs a virtual dual-attention task to stimulate their working memory and facilitate the reconsolidation of the traumatic memory trace. In ACET, the focus is on the active interactions between the patients and trauma-related virtual environment. The patients are expected to gain new corrective experiences by independently accomplishing missions in the trauma-related virtual environment by actively interacting with it (e.g., exploring and manipulating the virtual environment) (Kengne et al., 2018).

For the third part of the first research question (1) the results demonstrated that the manualization of the treatment protocols also varies according to the virtual trauma confrontations. Furthermore, the results showed that the VRET protocols are heterogeneous. In contrast, the virtual exposure treatment protocols used in all the 3MDR and ACET studies were the same. Most of the studies on VRET (70%) included 6–11 virtual exposure sessions, lasting 30–45 min. In the remaining studies, the virtual exposure sessions were conducted until the within-session habituation was attained. Therefore, the virtual exposure sessions at the beginning were longer (ca. 100 min) than the later ones (ca. 20 min). All the studies were based on the emotional processing theory; therefore, habituation was a prerequisite for treatment success. However, the difference in exposure duration raises the question of whether the predetermined time window in the 70% of the VRET studies was sufficiently long for the patients to experience habituation after the fear structure activation. Therefore, future studies should examine whether the duration of virtual exposure moderates the efficacy of VRET. Furthermore, the treatment protocols of VRET varied according to whether medication or at-home *in vivo* exposure exercises were included. This heterogeneity made the comparability of the VRET studies challenging. It is unclear whether the efficacy of VRET is attributable to the virtual visualization of the patient's recounting or other factors (e.g., exposure duration, medication, and at-home *in vivo* exposure exercises). In contrast, in the 3MDR studies, the same treatment protocols were used in the virtual exposure sessions, which were six, weekly sessions of 90 min. The procedure of the virtual exposure sessions was also standardized. Although the study designs of the two recent 3MDR RCTs were different (Bisson et al., 2020; Van Gelderen et al., 2020), the virtual trauma exposure was identical. Future systematic reviews can build on this and examine the efficacy of the virtual exposure sessions. In case of ACET, the same treatment protocol was used in both the single-case studies, which included two preparatory sessions and six indirect exposure sessions (Kengne et al., 2018; Menelas et al., 2018). These sessions consisted of several missions for the patients to accomplish. It was assumed that the new inhibitory experiences during the indirect exposure sessions would reduce the PTSD symptoms. However, there are only two single-case studies from the same authors. Therefore, it is necessary to interpret the results with caution.

Referring to the first part of the second research question (2), the results showed that HMDs were used in 12 studies, and CAVEs were applied in six. Detailed analyses revealed that 75% of the HMDs were released in 2005–2006, and the remaining 25% were released in 2012–2013. Therefore, the effects of technological advances on the efficacy or acceptance of virtual trauma interventions are currently unknown.

Regarding the second part of the second research question (2), the results showed that the software used in VRET, 3MDR, and ACET are also different. Pre-programmed virtual scenarios are used in VRET. The patients control their perspectives through head movements and navigate through the virtual environment using a gaming controller. Further active interactions, such as selecting and manipulating virtual objects, independently exploring the virtual environment, or interacting with avatars, are not intended. Therefore, it is unclear how this lack of interaction influences the sense of spatial or social presence (Sherman and Craig, 2019). Moreover, because the scenarios are preprogrammed, it is not possible to create a virtual trauma-related environment that completely matches the patient's recounting, thereby leading to incongruency. Therefore, breaks in the sense of spatial or social presence and plausibility may occur (Slater, 2009; Oh et al., 2019). In contrast, a completely unique virtual environment is used in 3MDR. For this purpose, individualized trauma-associated, fear-inducing, and avoided images related to the patient's traumatic experience are projected onto a CAVE. In this CAVE, the patient approaches the images, which simultaneously increase in size. However, in contrast to VRET, these are 2D representations without stereoscopy. As Cummings and Bailenson (2015) demonstrated in their meta-analysis, there is a medium positive correlation between stereoscopy and spatial presence. Similar to VRET, ACET uses computer-programmed virtual environments. However, it focuses on the active interaction between the patient and virtual environment. In addition to navigating and changing perspectives using head movements, it is intended that the patients explore the virtual environment independently; they can select and manipulate virtual objects and interact with artificial-intelligence-programmed road drivers. ACET integrates several factors to enhance the possibility of patients experiencing spatial and social presence.

Overall, the choice of hardware and software depends on the type of virtual trauma intervention. The advantages and disadvantages mentioned earlier can influence the sense of spatial and social presence, which is presumed to be a major influence on treatment success in all virtual trauma interventions. These differences are also relevant from a cost-efficiency perspective. As mentioned in the introduction, VRET and ACET have the advantage of low hardware costs. However, programming a virtual trauma-related environment is expensive and time-consuming. Additionally, a virtual trauma-related environment is always customized to one specific trauma type and population, limiting its generalizability. Table 1 supports this hypothesis, showing that the subjects of almost all VRET studies (90%) were US veterans with combat-related PTSD. In contrast, there are almost no software costs in 3MDR because it uses trauma-related images. However, the hardware costs for a CAVE are still high. Nevertheless, once a CAVE has been installed, 3MDR is more likely to be generalizable to different types of traumas and samples because there are almost no software costs.

Regarding the third research question (3), the results revealed that neither the spatial nor social presence was assessed in any of the 18 studies; thus, no information could be retrieved regarding the potential effect of spatial or social presence on the efficacy of virtual trauma interventions. This is a surprising result as all virtual trauma interventions (VRET, 3MDR, and ACET) are based on the assumption that the sense of presence is an important prerequisite. Therefore, it is not comprehensible why none of the selected studies has empirically examined it. This illustrates the need for future research to examine whether spatial presence constitutes a crucial mechanism in shaping the efficacy of virtual trauma interventions, as previously assumed by some researchers (Kothgassner et al., 2019).

Regarding the fourth research question (4), it was equally surprising that there is no empirical evidence on whether virtual trauma intervention is particularly effective for PTSD patients with imagination difficulties. For example, for VRET, it is assumed that virtual visualizations of the patient's recounting can particularly help patients who are unable or unwilling to imagine the traumatic event. However, this assumption cannot be verified because there is no empirical evidence of the usefulness of virtual trauma intervention for such patients. Therefore, future research is required to establish whether virtual trauma interventions are particularly effective for PTSD patients with imagination difficulties.

However, recent RCTs support the argument that virtual trauma interventions can reach patients who cannot benefit from established treatment guidelines (Bisson et al., 2020; Van Gelderen et al., 2020). The reason trPTSD patients benefit from 3MDR has not been examined, and the results should be interpreted with caution because they are not sufficiently confirmed, owing to small sample sizes. However, these results demonstrate that 3MDR can make clinically meaningful difference to the symptoms of trPTSD patients who do not benefit from imaginal trauma-focused guideline procedures. Based on these results, clinicians can weigh the acquisition costs of a CAVE against the long-term therapy costs for trPTSD patients. This could justify the acquisition costs for a CAVE and enhance the obtainability of 3MDR in future. It should be noted that such a cost-efficiency decision is currently possible only for 3MDR because there is no empirical evidence of the efficacy of VRET and ACET for trPTSD.

### Limitations

The findings of the present scoping review must be viewed critically based on various limitations. For example, the search was only conducted in English and German so we might have missed important studies written in other languages. In line with this, we found that the included studies were mostly conducted in English-speaking countries (55.5% in North America, 16.6% in Canada, 5.5% in the United Kingdom) and the remaining studies were conducted in the Netherlands. In order to counteract a potential country bias, pre-registration databases of various countries (Korea, China, Brazil, Cuba, Iran, Germany, Netherlands, EU clinical trials register, ClinicalTrials.gov and ISCRCTN registry) were screened to ascertain whether other countries are planning to or are currently examining virtual trauma interventions. A total of four pre-registrations were identified, one in the Netherlands, two in North America and one in Canada. These findings and the study characteristics of the 18 included studies suggest that there is a geographic pattern for virtual trauma interventions, which includes predominantly English-speaking countries and the Netherlands. However, given that our search is based on English and German keywords, a language bias cannot be ruled out so the findings should be interpreted with caution.

In addition we would like to point out that even though VRET was the most frequently published therapeutic virtual intervention for PTSD patients, and thus may be regarded a pioneer in the field, to date there are only results from the US, specifically from American war veterans. Also, the 3MDR studies focused on war veterans. Only the case studies on ACET examined PTSD following truck driver accidents. Hence, less is known about the efficacy of virtual trauma intervention for non-deployment related PTSD (e.g., domestic violence, natural disasters). Finally, patients in the included studies were predominantly male adults. Therefore, future research is needed to replicate the results with different populations (e.g., female, children, adolescents, and elderly adults) and with a broader range of traumatic events to confirm the generalizability of the findings.

## Conclusion

Previous studies have focused on the efficacy of VRET, which is one type of virtual trauma intervention. Therefore, this scoping review examined other virtual trauma interventions. We identified three different virtual trauma intervention approaches, namely, VRET, 3MDR, and ACET. These three approaches are based on different *in sensu* therapeutic frameworks. They vary in terms of transferring them into VR and of the treatment protocols. Although the procedures used for the virtual exposure in 3MDR and ACET are the same, we observed heterogeneity in VRET; therefore, we identified potentially confounding variables (exposure duration, medication, and at-home *in vivo* exposure) for the efficacy of VRET. Furthermore, our results demonstrated that the hardware and software are determined by the specific virtual trauma approach. From a cost-efficiency perspective, 3MDR seems promising. Although it has high hardware costs, two recent 3MDR RCTs demonstrated that it is efficient for trPTSD. Based on these results, clinicians can weigh the acquisition costs of a CAVE against the long-term therapy costs for trPTSD patients. Such a cost-efficiency decision currently applies only to 3MDRs because we found no empirical evidence of the efficacy of VRET and ACET for trPTSD.

We revealed other research gaps. Although all the studies posited that the sense of presence was an important prerequisite for treatment success, it was not examined in any of the studies included in this review. Therefore, we strongly recommend that future studies examine whether the sense of presence (spatial and/or social) influences treatment success. Moreover, it also remains unclear to what extent more recent technological advances relating to immersive technology have affected virtual trauma interventions because most of the HMDs utilized were released over 15 years ago. Furthermore, we did not identify any empirical evidence that virtual exposure is particularly helpful for patients with imaginations difficulties. This is surprising because it is a frequently cited argument for VRET. Thus, future research should examine whether virtual exposure can benefit PTSD patients with imagination difficulties. Finally, our descriptive results revealed that most studies examined male soldiers with combat-related PTSD. Therefore, replication studies with different trauma types and populations are warranted.

## Data Availability Statement

The original contributions presented in the study are included in the article/supplementary materials, further inquiries can be directed to the corresponding author/s.

## Author Contributions

TK, R-JG, HH, and HS designed the review. TK conducted the systematic searches and completed data extraction. TK and R-JG wrote the first draft of the manuscript. AF, OK, HH, and HS contributed extensively to the first draft and to the final manuscript. All authors read and approved the final manuscript prior to submission.

## Conflict of Interest

The authors declare that the research was conducted in the absence of any commercial or financial relationships that could be construed as a potential conflict of interest.

## Footnotes

^1^This includes, e.g., studies that examined virtual trauma exposure focusing on diagnostic aspects (Ridout et al., 2017; Van‘t Wout et al., 2017; Maples-Keller et al., 2019).

^2^In reviews and book chapters, the references were examined by hand to identify further publications.

^3^Difede et al. (2013) also reported that the virtual exposure sessions were conducted until with-session habituation was attained (50% reduction of the SUD). However, they also set a time limit of exposure (45 min).

^4^Supplementary Figures 1–3 contains the flow diagram on the pre-registration search.
