# Supplementary figures and images for: Virtual Trauma Interventions for the Treatment of Post-traumatic Stress Disorders: A Scoping Review
Source: Front Psychol. 2020 Nov 13;11:562506. doi: 10.3389/fpsyg.2020.562506 (PMC7691274; doi:10.3389/fpsyg.2020.562506)

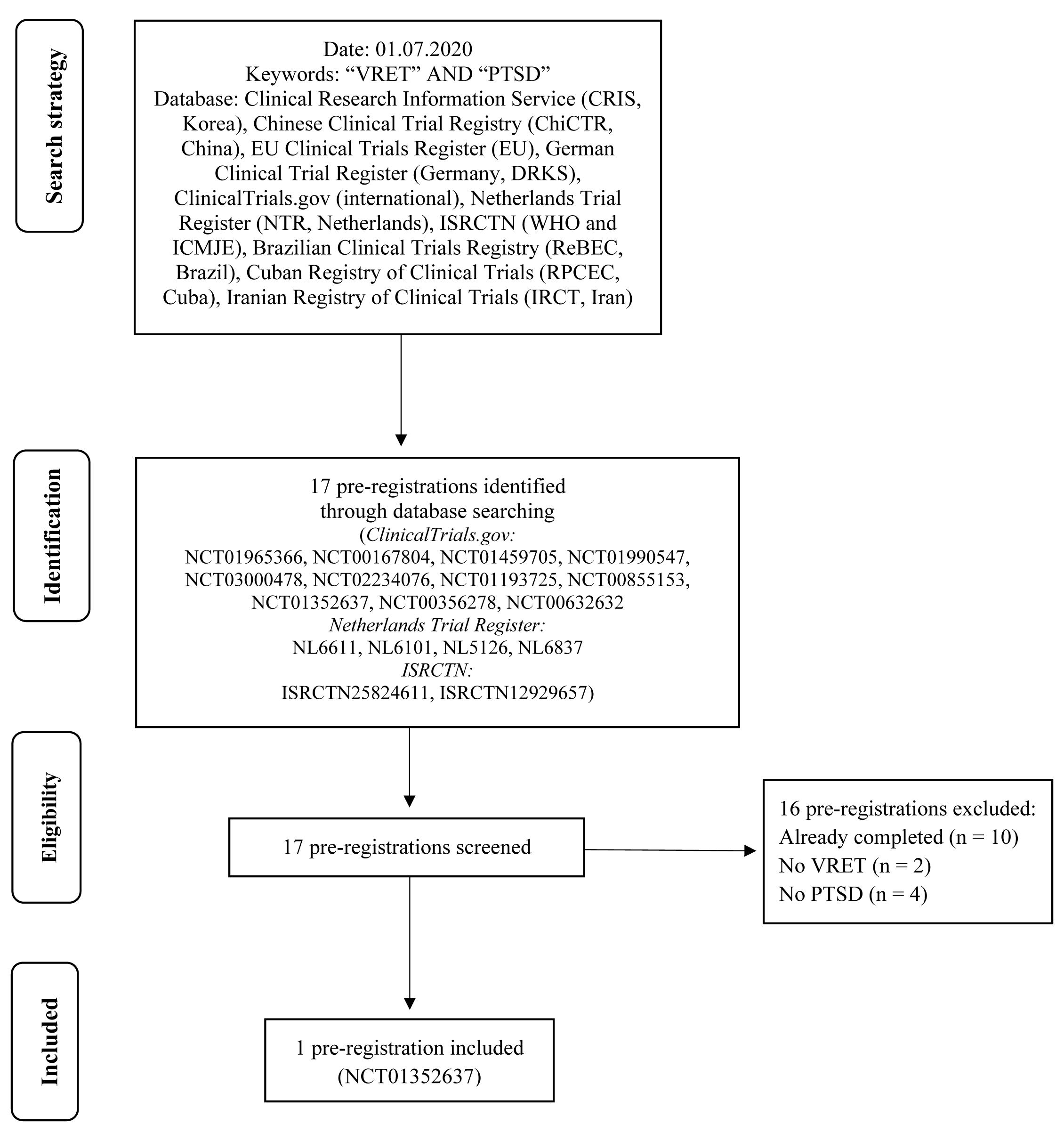

Supplement: Supplementary file 1 [file Image_1.TIF]

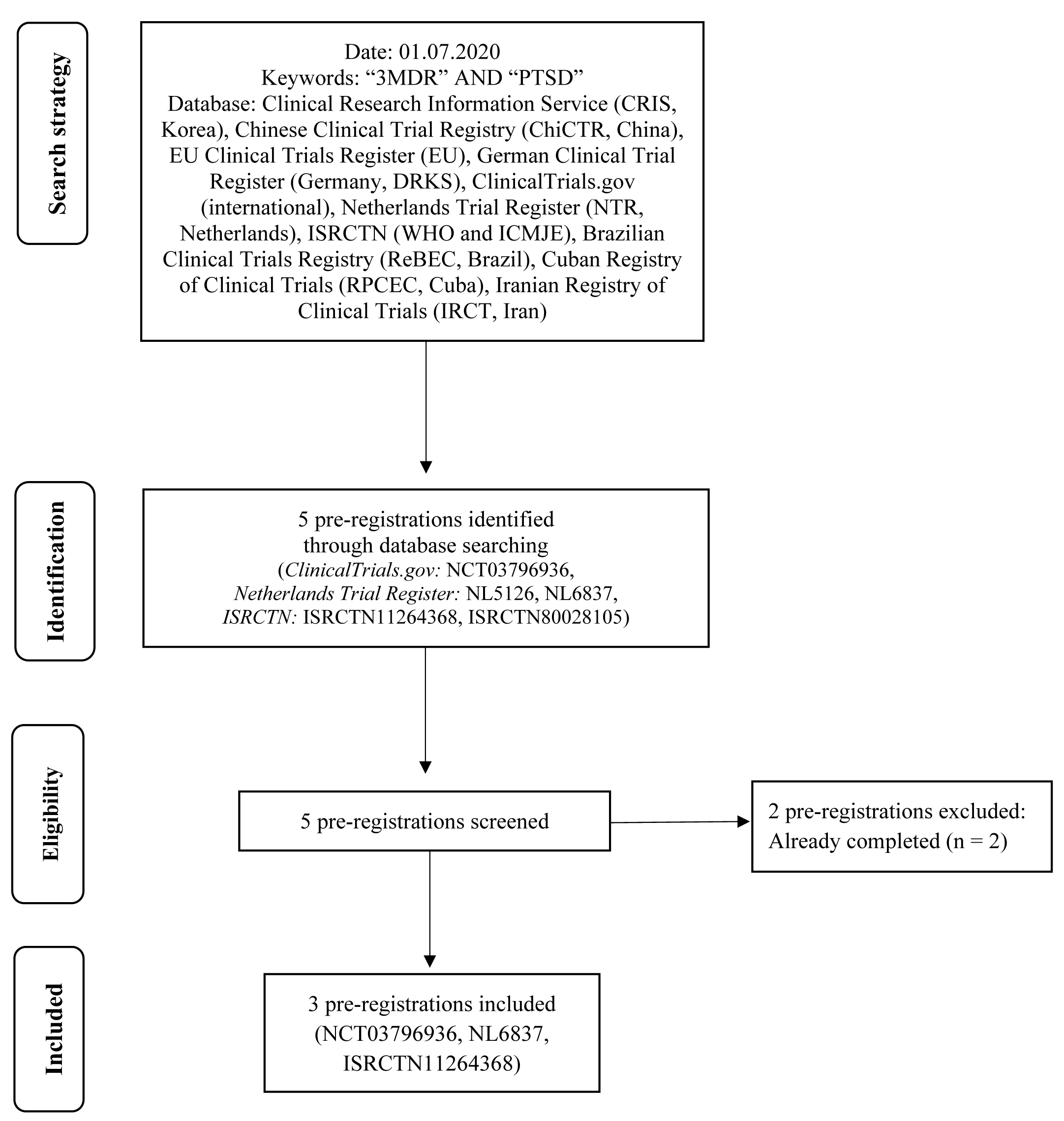

Supplement: Supplementary file 2 [file Image_2.TIF]

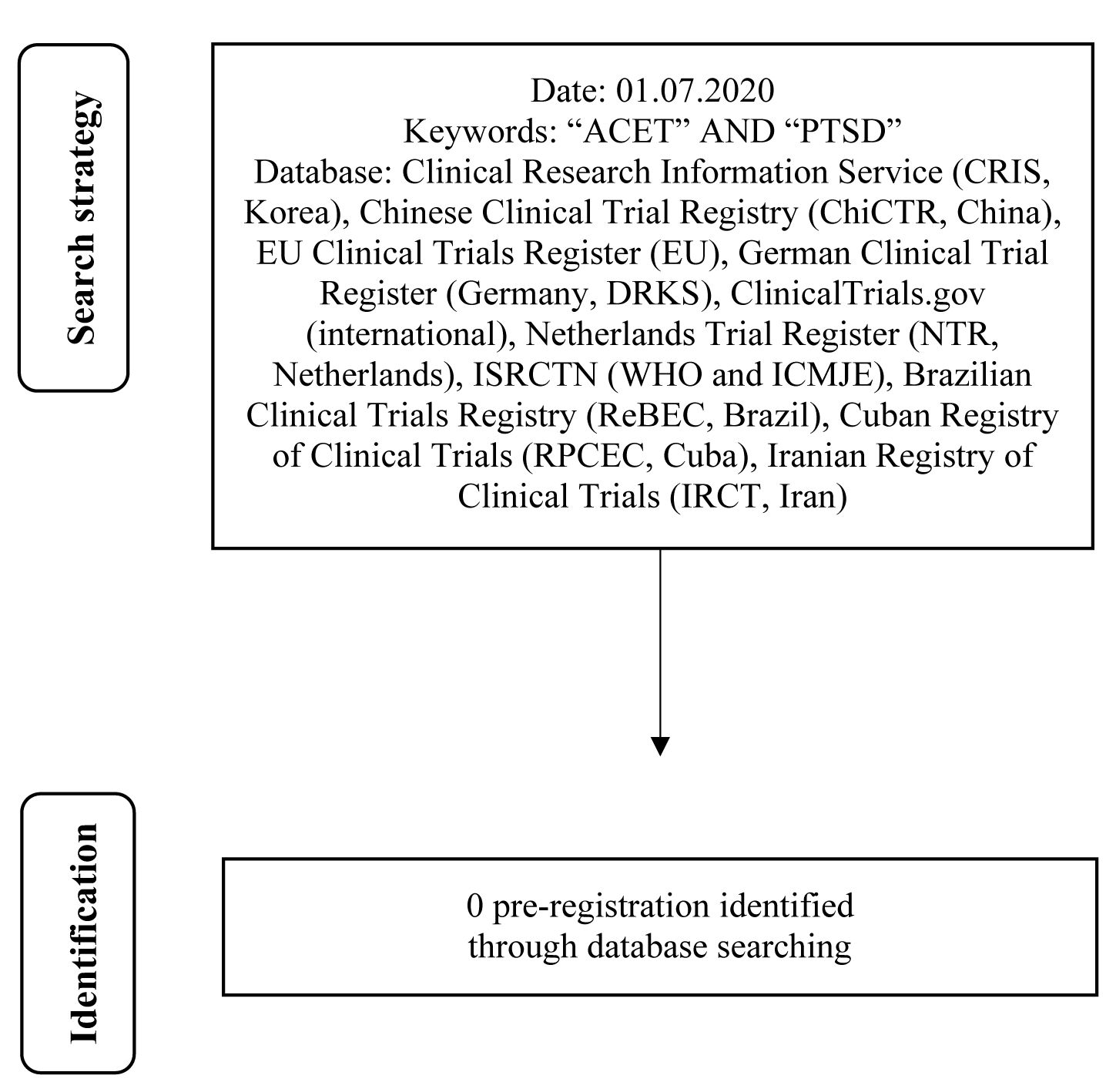

Supplement: Supplementary file 3 [file Image_3.TIF]
